# Recent Progress in Technetium-99m-Labeled Nanoparticles for Molecular Imaging and Cancer Therapy

**DOI:** 10.3390/nano11113022

**Published:** 2021-11-10

**Authors:** Sajid Mushtaq, Asia Bibi, Jung Eun Park, Jongho Jeon

**Affiliations:** 1 Department of Nuclear Engineering, Pakistan Institute of Engineering and Applied Sciences, P. O. Nilore, Islamabad 45650, Pakistan; sajidmushtaq@pieas.edu.pk; 2 Department of Chemistry, University of Wah, Rawalpindi, Punjab 47040, Pakistan; asiabibi.uow@gmail.com; 3 Department of Applied Chemistry, College of Engineering, Kyungpook National University, Daegu 41566, Korea; pje1204@knu.ac.kr

**Keywords:** technetium-99m, nanoparticle, molecular imaging, cancer therapy, radiolabeling

## Abstract

Nanotechnology has played a tremendous role in molecular imaging and cancer therapy. Over the last decade, scientists have worked exceptionally to translate nanomedicine into clinical practice. However, although several nanoparticle-based drugs are now clinically available, there is still a vast difference between preclinical products and clinically approved drugs. An efficient translation of preclinical results to clinical settings requires several critical studies, including a detailed, highly sensitive, pharmacokinetics and biodistribution study, and selective and efficient drug delivery to the target organ or tissue. In this context, technetium-99m (^99m^Tc)-based radiolabeling of nanoparticles allows easy, economical, non-invasive, and whole-body *in vivo* tracking by the sensitive clinical imaging technique single-photon emission computed tomography (SPECT). Hence, a critical analysis of the radiolabeling strategies of potential drug delivery and therapeutic systems used to monitor results and therapeutic outcomes at the preclinical and clinical levels remains indispensable to provide maximum benefit to the patient. This review discusses up-to-date ^99m^Tc radiolabeling strategies of a variety of important inorganic and organic nanoparticles and their application to preclinical imaging studies.

## 1. Introduction

Cancer is a considerable cause of mortality and morbidity in every region of the world, irrespective of medical facilities, human development, and scientific research. According to the GLOBOCAN survey, a total of 19.3 million new cancer cases and more than 10 million deaths from various cancer types were registered in 2020 [1]. Over the past decades, preclinical molecular biology research has provided tremendous results in the exploration of cancer at the cellular level. However, only a few experimental strategies have passed the rigorous clinical trial process to benefit cancer patients in clinics. One of the major obstacles is the lack of availability of facilities and tools to understand different molecular events inside patients [2,3]. The strong demand for the *in vivo* imaging of different biological events in patients has brought a revolution in molecular imaging. Molecular imaging is a single or combination of various modalities for the non-invasive visualization, characterization, monitoring, and measurement of various biological events at the cellular level in cancer patients [4]. Previously, X-rays [5], ultrasound [6], computed tomography (CT) [7], optical fluorescence [8], magnetic resources imaging (MRI) [9], single-photon emission computed tomography (SPECT) [10], and positron emission tomography (PET) [11] have been explored for cancer diagnosis and to monitor the efficacy of ongoing treatment procedures. Preclinical and clinical data reveal that each molecular imaging system has its specific utilization, strengths, and limitations (Table 1). For instance, MRI images give high spatial resolution, but their low sensitivity is their main limitation. In contrast, radioisotope-aided imaging modalities (SPECT and PET) have extremely high sensitivity and unlimited penetration depth, but show poor spatial resolution [12]. Optical imaging is an excellent non-invasive image technique, but its low depth penetration limits its clinical applications in several cases. Therefore, various imaging technologies have been considered to combine the advantages of different modalities and minimize the limitations. These combinations of imaging facilities, called multimodality imaging, provide better and more comprehensive data regarding disease and ongoing medication and treatment procedures [13]. Multimodality imaging systems such as SPECT/CT, SPECT/MRI, PET/CT, and PET/MRI are now commercially available, and rigorous research is continuing to improve their quality [14].

Similarly, therapeutic drugs loaded on different platforms are being monitored using radioisotopes and multimodality imaging systems. The development of new contrast agents such as radiotracers and radiopharmaceuticals is an integral part of the success of multimodality imaging and cancer therapy. Nanotechnology can provide an excellent platform for developing novel tracers to support these hybrid multimodality imaging systems [15]. Nanomedicine has already shown excellent results in diagnostics, gene therapy, drug delivery, and other vital areas of development and clinical research [16]. Nanoparticles (NPs), essential elements of nanotechnology, are materials with an overall size of fewer than a hundred nanometers, typically smaller than human cells and comparable to the size of biomolecules such as antibodies, proteins, and receptors enzymes. Because of this distinctive feature, various well-designed NPs demonstrate long blood circulation, easy and efficient interaction with biological molecules on the target cell surface and cell penetration ability [17]. For instance, NPs with typical dimensions ranging from 20 to 100 nm showed enhanced permeability and retention (EPR) effects; they can accumulate at target areas closer to cancer cells. Additionally, NPs possess a high surface area to volume ratio; with this ability, they provide an excellent platform to load a variety of cancer-targeting precursors, therapeutic drugs, imaging, and therapeutic radioisotopes [18].

The radiolabeling of NPs provides a new class of radiotracers for radiotherapy, PET, or SPECT-based imaging. Radiolabeled NPs provide excellent information regarding the pharmacokinetics, biodistribution, and efficiency of the therapeutic drugs delivered to the cancer cell. Therefore, cancer treatment and imaging using radiolabeled NPs holds great potential for preclinical research and clinical applications [19]. This review discusses the current strategies and prospects of ^99m^Tc labeled NPs for widely available SPECT system-based imaging. This article consists of two categories. The first part describes the ^99m^Tc radiolabeling of inorganic nanoparticles and the second provides information on the preparation and applications of ^99m^Tc radiolabeled organic nanoparticles. In addition, the main focus of discussion is radiolabeling strategy and surface modifications, and their impact on the stability and pharmacokinetics of radiolabeled products.

## 2. Radiolabeling of Nanomaterials

A variety of radiolabeled nanoparticles have been developed as useful radiotracers, and these materials, such as gamma camera or SPECT imaging (Indium-111, Technetium-99m, Iodine-125, etc.), PET imaging (Flourine-18, Zirconium-89, Copper-64, Gallium-68, etc.), radioisotope therapy (Copper-67, Lutetium-177, Astatine-211, Actinium-225, Yitrium-90, etc.) and dual modality imaging have been applied to nuclear medicine. The selection of an appropriate nanomaterial, radioisotope and radiolabeling strategy depends upon the intended medical application. To introduce a radioisotope to a functional nanomaterial, several methodologies were developed and used in preclinical studies. The surface modifications of a nanomaterial and radiolabeling strategies have great impact on the stability of a radiotracer and its pharmacokinetics *in vivo*. Herein, technitium-99m, one of the most widely used radioisotope in the field of nuclear medicine has been selected for detailed discussion.

## 3. Radiolabeling of Nanomaterial Using ^99m^Tc

### Technetium-99m (^99m^Tc) Properties and Production

**Figure 1:**
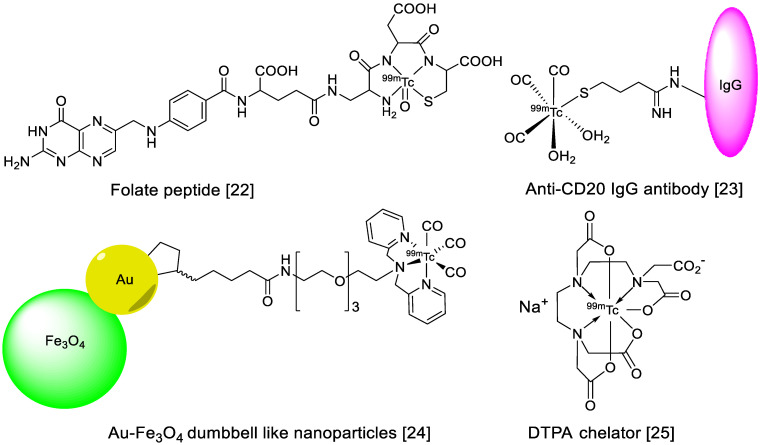
Examples of ^99m^Tc radiolabeled biomolecules and nanoparticles [22,23,24,25].

After the introduction of positron emission tomography (PET), it was assumed that the more advanced technology would have replaced the old SPECT technology and SPECT-based radiopharmaceuticals. Interestingly, technetium-99m (^99m^Tc)-based radiopharmaceutical production continues, and the number of clinical examinations using ^99m^Tc and SPECT is still greater than those using PET-based molecular imaging [20]. Despite its great success, PET is still a relatively expensive technology, and the production and radiolabeling of PET radioisotopes are cumbersome tasks in some developing nations. Therefore, ^99m^Tc is a globally recognized radioisotope, and a major component of day-to-day nuclear imaging procedures. There are many reasons behind this success, such as the low cost and straightforwardness of SPECT procedures, the easy availability of ^99m^Tc in the form of ^99^Mo/^99m^Tc generators, and the worldwide commercial availability of ^99m^Tc labeling kits. Moreover, its distinguished nuclear properties include a suitable half-life (6.0 h), isomeric decay to a ground state, monochromatic gamma-ray emission (140.5 keV, 98.5%), highly stable coordination chemistry, and high-quality SPECT images make it an ideal radioisotope in the field of nuclear medicine [21]. ^99m^Tc is eluted from ^99^Mo/^99m^Tc generators in the form of pertechnetate (TcO_4_^−^), and this is the best strategy to obtain ^99m^Tc in the clinic. ^99^Mo is mainly produced in research reactors by irradiating molybdenum oxide or metallic molybdenum, or by irradiating uranium-235 with thermal or fast neutrons. During a fission reaction, ^99^Mo is obtained with other fission products as impurities, and many radiation shielding and purification steps are required to obtain pure parent radionuclide. However, little processing is required for the conversion of ^98^Mo to ^99^Mo. In a typical ^99^Mo/^99m^Tc generator, approximately 20 mg of ^99^Mo are absorbed on an alumina column in the form of ^99^MoO_4_^2−^. Then, 1.0 M saline is used to elute pure ^99m^Tc in every 24 h. The direct or chelator-based radiolabeling of amino acids, peptides, proteins, antibodies, and organic or inorganic NPs (Figure 1) has demonstrated, valuable results at the preclinical and clinical levels.

## 4. Inorganic Nanoparticles

Inorganic NPs such as iron oxide, gold, silver copper, cobalt, titanium, silica, manganese gadolinium, quantum dots, and silicon have been extensively used in nanomedicine. Inorganic NPs possess unique size and material-dependent physiochemical properties which are not available in other traditional polymer and lipid-based NPs. Moreover, some unique features such as size-controlled synthesis, easy surface functionalization, and *in vitro* and *in vivo* stability in various media make inorganic NPs an excellent platform to synthesize multimodality imaging and therapeutic agents. ^99m^Tc-radiolabeled nanoparticles are summarized in Table 2.

### 4.1. Iron Oxide Nanoparticles

**Figure 2:**
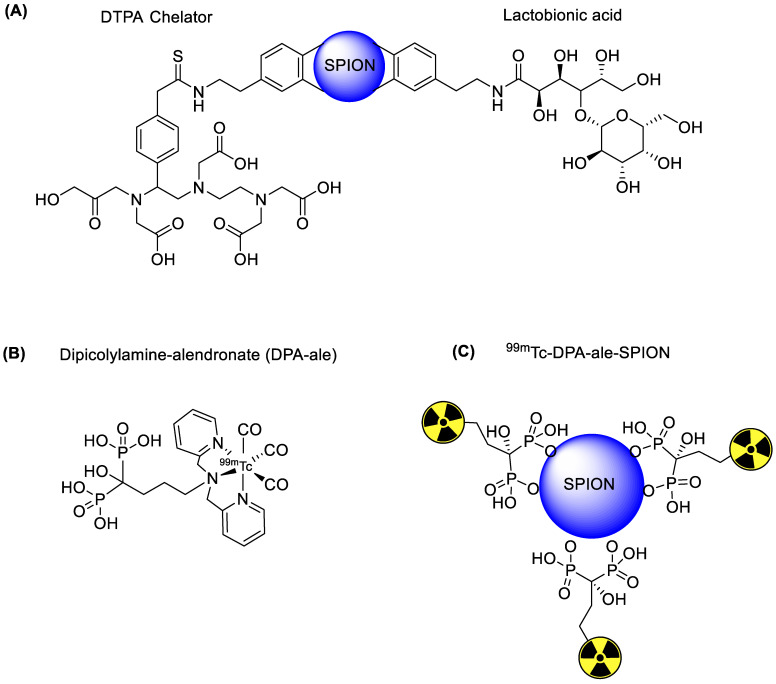
^99m^Tc radiolabeling of various SPIONs. (**A**) Schematic of a lactobionic acid-installed SPION [28]. (**B**) ^99m^Tc radiolabeled DPA-ale. (**C**) ^99m^Tc radiolabeled DPA-ale-installed SPION [29].

**Figure 3:**
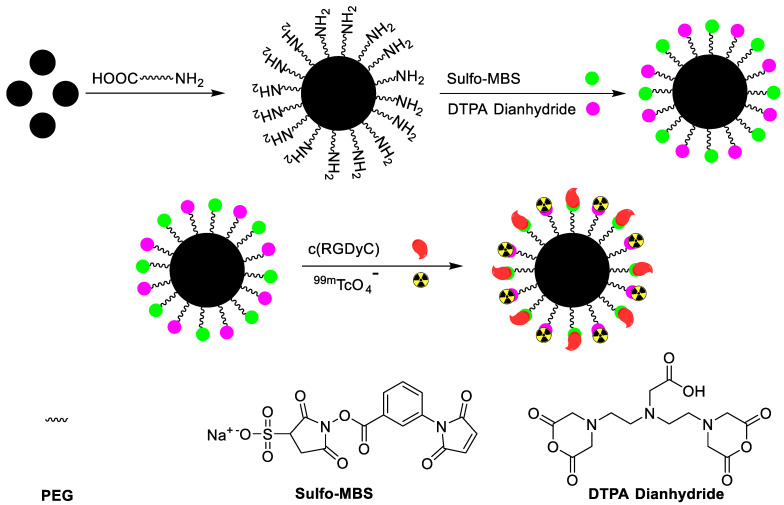
Procedure for the synthesis of c(RGDyC) decorated iron oxide nanoparticles and radiolabeling with ^99m^Tc [31].

Iron oxide NPs are classified into three categories depending upon size, such as monocrystalline iron oxide (10–30 nm), ultra-small superparamagnetic iron oxide (5–10 nm), and standard superparamagnetic iron oxide (60–150 nm). Among these iron oxide NPs, standard superparamagnetic iron oxide has been extensively used because of some unique biocompatibility, facile surface functionalization, and enhanced magnetic contrast [26]. Standard superparamagnetic iron oxide NPs can become trapped in the reticuloendothelial system through phagocytosis or endocytosis. However, standard superparamagnetic iron oxide NPs are not harmful to the human body, and can be dissolved under acidic conditions. The resulting iron can be retained in the human body, which has enough iron storage capacity (3–5 g per human adult). Moreover, the toxicity of iron oxide NPs can be avoided by using an organic coating such as synthetic polymers, dextran, or other biocompatible materials. Iron oxide NPs based on single or dual imaging are further coated with tumor-specific biomolecules to increase target accumulation. In the hyperthermia treatment procedure, iron oxide NPs are introduced inside a body and an alternative magnetic field is applied near the tumor area after enough iron oxide NPs have been introduces. The NPs convert magnetic energy into heat energy to damage the cancer cell. In order to assess the efficiency of the hyperthermia procedure, iron oxide NPs were radiolabeled with ^99m^Tc to confirm maximum accumulation at the tumor area. In a typical procedure, the iron oxide NPs were treated with ^99m^TcO_4_^−^ in the presence of tetrahydroborate exchange resin as a reducing agent. The ^99m^Tc was incorporated on the surface of iron oxide NPs via a redox reaction. The radiolabeled NPs showed high *in vitro* stability. However, the radiolabeled NPs were not subjected to *in vivo* testing [27]. ^99m^TcO_4_^−^ does not form a complex with iron oxide NPs in the absence of a reducing agent. Interestingly, a minute amount of stannous chloride dehydrate is enough to reduce pertechnetate to the lower oxidation state and form a stable complex with iron oxide NPs. Stannous chloride-mediated ^99m^Tc labeled iron oxide demonstrated high stability under physiological conditions, and maximum radioactivity was observed in the liver. In 2009, ^99m^Tc labeled superparamagnetic iron oxide NPs were synthesized for the dual (magnetic resonance and SPECT) imaging of hepatocytes. The superparamagnetic iron oxide NPs were synthesized, and the surface modification was performed using dopamine. The dopamine arm was used to attach lactobionic acid to the target hepatocyte receptor and 2-(4-Isothiocyanatobenzyl)-diethylenetriamine-pentaacetic acid (DTPA) as ^99m^Tc chelator (Figure 2). The lactobionic acid on the surface of the iron oxide NPs efficiently targets hepatocyte receptors in the liver. Both magnetic resonance and SPECT showed high accumulation of the tracer in the target area. The images showed high spatial resolution and sensitivity in the final results. The biodistribution results supported the image data and confirmed that the probe could be used as a dual imaging agent against liver diseases [28]. Iron oxide is an excellent platform for drug loading, and radioisotopes provide an excellent NP surface coating material. Poor surface coating sometimes results in the decomposition of the complex over time, and loss of both the drug and the radioisotope. The literature reveals that phosphonates, monosulfonates, monocarboxylates, citrates, and catechol can form a stable coating on the surface of iron oxide NPs. However, it has been observed that the redox reaction of catechol under a protic environment leads to the precipitation and decomposition of NPs. Interestingly, the development of biocompatible, nonpolymeric methods, such as bisphosphonate-based coating of iron oxide NPs, provides a solution. Recently, a clinically approved magnetic resonance imaging superparamagnetic iron oxide (Endorem) was coated with bisphosphonate chelator (Figure 2). A ^99m^Tc tricarbonyl kit was used to synthesize radiolabeled Endorem. Initially, the bisphosphonate chelator was radiolabeled with ^99m^Tc in high radiochemical yield (>95%) and radiochemical purity. In the next step, a ^99m^Tc chelator was incorporated on the surface of Endorem at elevated temperature. ^99m^Tc radiolabeled NPs were obtained via bisphosphonate binding on the surface of NPs. The radiolabeled NPs showed excellent *in vivo* and *in vitro* stabilities. SPECT/CT and magnetic resonance imaging simultaneously confirmed the presence of tracer, predominantly in the liver and spleen. This dual imaging agent provided magnetic resonance imaging with high spatial resolution, and at the same time, the SPECT/CT data provided crucial quantitative information [29]. The surface modification of iron oxide NPs plays a vital role in reducing toxicity under physiological conditions. Surface coating with biocompatible materials such as aspartic acid, dextran, polyethylene glycol, and dimercaptosuccinic acid (DMSA) increases cell entrance and decreases the toxicity effects. The ^99m^Tc radiolabeling of DMSA coated iron oxide NPs demonstrated interesting results [30]. DMSA provides an anionic coating on the surface of NPs, which prevents the aggregation of blood proteins on the surface of NPs. Additionally, it increased cell absorption and decreased the removal of NPs by the reticuloendothelial system. The DMSA-based radiolabeling of NPs provided better *in vitro* and *in vivo* stability, minimum toxicity, and efficient quantitative information regarding the accumulation of iron oxide NPs in the liver and spleen. Iron oxide NPs have shown great potential for treating liver diseases. Additionally, they provide an excellent platform by which to install tumor-specific biomolecules. Recently, c(RGDyC) peptide-installed iron oxide NPs were synthesized to target α_v_β_3_-positive tumor cells and tumor angiogenesis. In addition, iron oxide NPs were also radiolabeled with ^99m^Tc to make a dual-modal SPECT/MRI imaging agent (^99m^Tc-SPION-RGD). NH_2_PEG-modified iron oxide NPs were sequentially treated with c(RGDyC) peptide, the chelating agent DTPA and ^99m^Tc to synthesize ^99m^Tc-SPION-RGD (Figure 3). ^99m^Tc-SPION-RGD showed excellent *in vivo* and *in vitro* selectivity towards H1299 α_v_β_3_-positive cells. Moreover, radiolabeled NPs provided MRI images with high spatial resolution. SPECT images provided quantitative data for liver uptake. ^99m^Tc radiolabeled iron oxide NPs have become an irreplaceable emerging tracer for dual imaging, and research is being conducted to improve image quality and sensitivity [31]. The *in vivo* aggregation of NPs has been exploited to improve image sensitivity. *In vivo* aggregation of NPs has some clear advantages, such as low dose of contrast agent, prolonged retention of tracers at the tumor site, and an enhanced *T*_2_ effect that improves MRI image quality. c(RGDyC)-installed, glutathione (GSH) sensitive ^99m^Tc-labeled iron oxide NPs have been synthesized. The aggregation of NPs was observed due to the presence of GSH in the tumor microenvironment. The *in vivo* aggregation of the tracer improved the sensitivity of MRI and SPECT images. The results were threefold better than normal c(RGDyC) containing iron oxide NPs [32]. Although iron oxide NPs are the most widely used drug carriers due to their biocompatibility, researchers have also attempted other combinations to synthesize superparamagnetic NPs. Recently, cobalt ferrite (CoFe_2_O_4_) NPs were radiolabeled with ^99m^Tc to explore their biodistribution and pharmacokinetics. In this study, *in vitro* and *in vivo* stabilities, biodistribution, and SPECT images of ^99m^Tc labeled Fe_3_O_4_ and CoFe_2_O_4_ were compared in normal mice. Direct radiolabeling of both hybrid NPs provided highly stable radiolabeled tracers with high radiolabeling yield and purity. Interestingly, both NPs had nearly similar biodistribution profiles. A high accumulation of radiotracers was observed in the liver and spleen [33]. Similarly, in 2015, R. Alberto et al. described the synthesis, ^99m^Tc radiolabeling, and *in vitro* stability of Au-Fe_3_O_4_NPs (core-shell and dumbbell) [24].

### 4.2. Gold Nanoparticles

**Figure 4:**
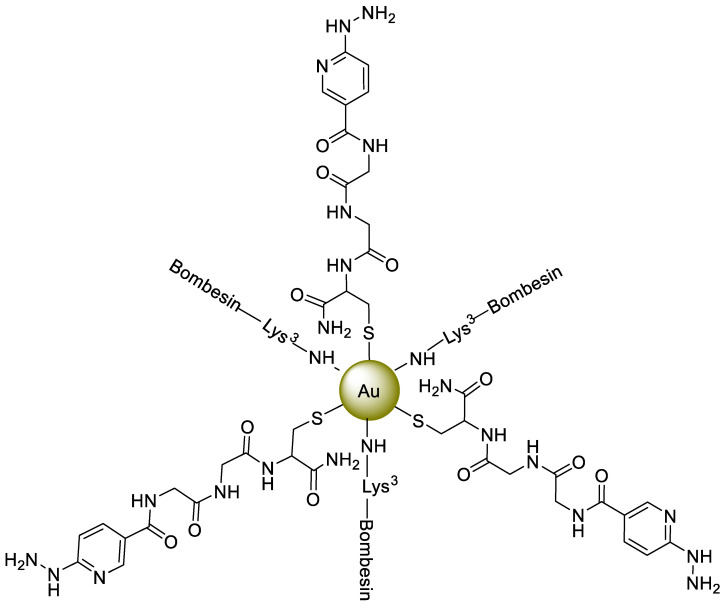
A HYNIC-GGC-AuNP-Lys^3^-bombesin core for ^99m^Tc radiolabeling [35].

Gold nanoparticle (AuNPs)-based contrast agents (MRI or CT) and imaging agents (PET or SPECT) have been extensively studied because of their unique properties, such as surface passivation, simple surface modification, optical properties, and biocompatibility. The first AuNP-based radiotracer was reported in 2004. Over the next decade, comprehensive investigations were performed to optimize radiolabeling procedures, surface chemistry, and pharmacokinetics studies [34]. The surface modification of NPs using tumor-specific peptides can increase image sensitivity. These therapeutic or imaging peptides can target over-expressed peptide receptors in the tumor cells. The over-expressed receptors on the surfaces of cancerous cells represent promising targets for diagnosis or therapy. (GRP-r), the peptide receptor that releases gastrin, is overexpressed in prostate cancer, breast, and metastatic lymph nodes, while a peptide Lys^3^-bombesin binds with GRP-r in high affinity. A multifunctional system was developed, comprised of 20 nm AuNPs having conjugation with Lys^3^-bombesin and HYNIC-Gly-Gly-Cys-NH_2_ [HYNIC (hydrazinonicotinamide), GGC(Gly-Gly-Cys) peptide] and radiolabeled with ^99m^Tc. In this multi-model system, HYNIC served as a ^99m^Tc chelator with a -Gly-Gly-spacer, and interactions with the AuNP surfaces were developed by a -Cys- NH_2_functinal group (Figure 4). TEM images and spectroscopic techniques reflected the interaction and successful binding of AuNPs with peptide and Lys^3^-bombesin. Afterwards, ^99m^Tc radiolabeling was successfully performed via the HYNIC-GGC ligand by employing the co-ligands EDDA/tricine and SnCl_2,_ and a radio-chromatogram confirmed more than 95% radiochemical purity. *In vitro* studies revealed that for overexpressed GRP-rin PC-3 cells, ^99m^Tc-AuNP-Lys^3^-bombesin exhibits higher specificity with 71% enhanced cellular uptake compared ^99m^Tc-AuNPs, while *in vivo* SPECT/CT imaging of this novel multifunctional system displayed tumor uptake of 6.39 ± 0.83% IA/g at one h with significant uptake by spleen and liver. However, the inability of ^99m^Tc-AuNP-Lys^3^-bombesin to cross narrow blood vessels resulted in significant accumulation in cancerous cells [35]. As per the sentinel lymph node (SLN) concept, this node or its groups are drain cancer. In the light of the above assumption, a cancer-free sentinel node is a strong indication of the non-spreading of the tumor. So, the design of a radiolabeled drug composed of ^99m^Tc based colloids with a size of less than 100 nm with the hydrazinonicotinamide (HYNIC) chelator is a practical approach for peptide radiolabeling to attain enhanced specificity and favorable pharmacokinetics, making such a system a potential imaging tool. A multifunctional model of ^99m^Tc-labeled AuNPs was stated in which the average size of AuNPs was around 20 nm. In this design, HYNIC served as a ^99m^Tc chelator, and interactions with the surface of AuNPs were developed with Cys-NH_2_ via a spacer (-Gly-Gly-). Afterward, two radiolabeled conjugates: (i) ^99m^Tc-EDDA/HYNIC-GGC-AuNP (^99m^Tc-AuNP) and (ii) ^99m^Tc-EDDA/HYNIC-GGC-AuNP mannose (^99m^Tc-AuNP-mannose) were prepared, and a radio-chromatogram confirmed more than 95% radiochemical purity. TEM images and spectroscopic techniques mirrored the interaction and successful binding of AuNPs with the peptide and the mannose. *In vitro* studies showed a 40% higher uptake of ^99m^Tc-AuNP-mannose compared to ^99m^Tc-AuNP. Moreover, *in vivo* studies also reflect enhanced uptake of ^99m^Tc-AuNP-mannose by the first lymphatic node (11.58 ± 1.98% ID at 1 h) compared to ^99m^Tc-AuNP (4.28 ± 1.49% ID at 1 h), thus confirming the specificity and targeted binding of ^99m^Tc-AuNP-mannose with overexpressed mannose receptors with minimum accumulation in the kidney and insignificant uptake by other tissues. Moreover, the clearance constant (λ) was found to be 0.1527 ± 0.0086 h^−1^ and 0.1405 ± 0.0047 h^−1^ for ^99m^Tc-AuNP-mannose and ^99m^Tc-AuNP, respectively [36].

**Figure 5:**
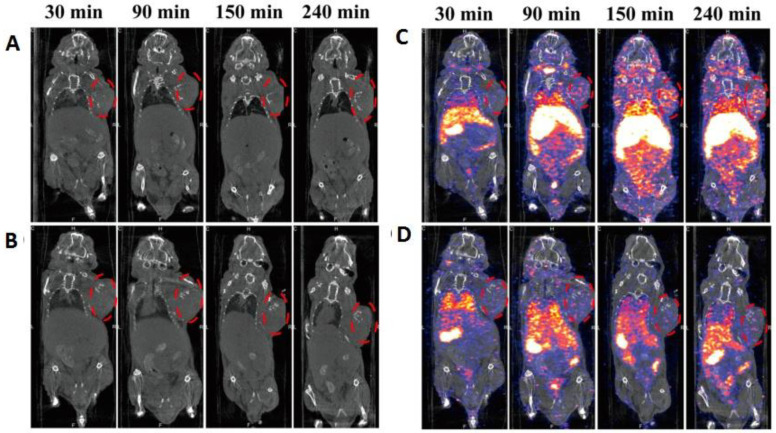
(**A**,**B**) *In vivo* CT and (**C**,**D**) SPECT/CT images of a xenografted tumor model after intravenous injection of {(Au^0^)_6_-G2-DTPA (^99m^Tc)-PEG-FA} DENPs (**A**,**C**, respectively) and ({(Au^0^)_6_-G2-DTPA (^99m^Tc)-mPEG} DENPs) (**B**,**D**, respectively). High uptake in the liver, spleen, and tumor is visible. Reprinted with permission from [40], with permission from American Chemical Society, 2016.

The angiogenesis process involves the sprouting of existing vessels, followed by the growth of new blood vessels. Thus, it is considered to be the prerequisite for the growth of tumors and metastasis. Overall, this process is controlled by integrins, also known as cell adhesion receptors. On the surface of normal endothelial cells, α_v_β_3_ integrin is barely expressed, but it tends to obtain overexpressed at tumor sites. Arg-Gly-Asp (RGD)-based radiolabeled peptides, particularly with a cyclic RGD (cRGD) sequence, possess selectivity and strong affinity for the α_v_β_3_ integrin, owing to enhanced multivalent sites, thus, making them potentially a radiopharmaceutical for imaging techniques involving non-invasive tumor angiogenesis monitoring. Following this key point, a system of ^99m^Tc-labeled AuNPs was developed in which the average size of AuNPs was found to be around 20 nm followed by conjugation to two peptides, HYNIC-GGC and cyclic[Arg-Gly-Asp-Phe-Lys(Cys)] {c[RGDfK(C)]}. The ^99m^Tc labeled peptides HYNIC-GGC and c[RGDfK(C)] were prepared then added to a AuNPs solution (AuNPs/HYNIC-GGC:AuNPs/c[RGDfK(C)] = 1:1250) to yield HYNIC-GGC-AuNP-c[RGDfK(C)] via interactions between -Cys- functionality and the AuNPs’ surfaces. Afterwards, ^99m^Tc-AuNP-RGD (^99m^Tc-EDDA/HYNIC-GGC-AuNP-c[RGDfK-(C)]) and ^99m^Tc-AuNP (^99m^Tc-EDDA/HYNIC-GGC-AuNP) with radiochemical purity of 96 ± 2% were found. TEM and spectroscopic techniques confirmed the successful functionalization of AuNPs with peptides via the thiol moiety. In human serum, ^99m^Tc-AuNP-RGD showed stability for more than 24 h, and *in vitro* studies in C6 cells confirmed the recognition of α_v_β_3_ integrins with cellular uptake 2.3 times higher than ^99m^Tc-AuNP. Micro-SPECT/CT imaging for ^99m^Tc-AuNP-RGD also reflected higher tumor uptake than ^99m^Tc-AuNP, making it a potential imaging tool for positive α_v_β_3_ patients. Moreover, the administration mode of the drug (either i.p. or i.v.) was found to significantly affect uptake by liver and spleen, which is decreased by switching administration mode from i.v. to i.p. [37]. The surface of cells’ reversible and strong binding between receptors and peptides is governed by the key principle of multivalency that assists in regulating numerous cellular activities. Hence, to achieve a target specified molecular recognition with a stable and biocompatible multimeric system, conjugation between AuNPs and peptides is a well-known strategy. Along this line, receptor-specific multimeric systems for kit formulation were prepared by employing ^99m^Tc labeled AuNPs followed by conjugation with either Lys^3^-bombesin, thiol-mannose or cyclo[Arg–Gly–Asp–D–Phe–Lys–(Cys)] c[RDGfK(C)] with radiochemical purity of 96 ± 2%. All three radiopharmaceuticals, ^99m^Tc-AuNP–Lys^3^-bombesin, ^99m^Tc-AuNP–mannose and ^99m^Tc-AuNP–c[RDGfK(C)] were found to exhibit suitable properties for be employment as potential target-specific agents for the molecular imaging of GRP-r-positive tumors, SLN detection, and tumor α_v_β_3_ expression, respectively. For GRP-r and α_v_β_3_ positive cells, ^99m^Tc-AuNP–Lys^3^- bombesin and ^99m^Tc-AuNP–c[RGDfK(C)] uptake were 1.8 and 2.2 times higher than ^99m^Tc-AuNP, respectively. Moreover, the kit showed excellent stability for six months when kept at 48 °C [38]. Bombesin and c[RGDfK(C)]-decorated AuNPs demonstrated excellent *in vitro* and *in vivo* results; however, these tracers are constrained in the cell membrane and cytoplasm. HIV Tat (49–57) peptide can penetrate the cell and reach the DNA. Moreover, a combined bombesin and Tat (49–57) peptide-based ^99m^Tc-labeled radiotracer showed an ability to penetrate prostate and breast cancer cells. Owing to their large surface area, AuNPs were decorated with bombesin and Tat (49–57) peptides to increase the stability of peptides and the retention time of the tracer near the cell microenvironment. The tracer was further radiolabeled with ^177^Lu via a DOTA chelator for radiotherapy of the cancer cell. An additional HYNIC chelator was incorporated for ^99m^Tc radiolabeling and *in vivo* monitoring of the radiotracer. The radiotracer was initially tested against PC3 human cancer cells to provide *in vitro* plasmonic photothermal therapy and targeted radiotherapy. The radiotracer showed specific accumulation in PC3 cells, which was 52.5% higher than ^99m^Tc radiolabeled AuNPs. Plasmonic photothermal therapy significantly reduced PC3 cell viability. Similarly, combined plasmonic photothermal and radiotherapy inhibited PC3 cell proliferation significantly more than AuNPs alone [39]. The radiolabeling of AuNPs largely depends upon the type of carrier system. A suitable carrier system not only provides radiolabeling stability but also influences the pharmacokinetics of the tracer. Organic macromolecules such as poly (amidoamine) (PAMAM) dendrimers with appropriate monodispersity are an excellent carrier upon which to load imaging agents. Dendrimer-stabilized AuNPs serve as a CT contrast agent for blood pool or tumor imaging. Recently, dendrimer-stabilized, folic acid-containing gold nanoparticles were radiolabeled with ^99m^Tc to obtain dual SPECT/CT imaging. The DTPA chelator was attached to the PAMAM dendrimer for the stable radiolabeling of gold nanoparticles. In an *in vitro* experiment, the radiotracer({(Au^0^)_6_-G2-DTPA(^99m^Tc)-mPEG} DENPs) showed high uptake in HeLa-LFAR cells. CT images showed contrast enhancement, and at the same time, SPECT images were obtained with high sensitivity (Figure 5). For *in vivo* experiments, the radiotracer was intravenously injected into the xenografted tumor model. High tumor uptake was observed. Moreover, high radioactivity was found in the liver and spleen due to RES clearance. The radiotracer was retained in the tumor for more than 24 h [40].

Resveratrol (Res) is a well-known phenolic compound owing to its cancer prevention and antioxidant properties which, upon tagging with a radionuclide, help it serve as a tumor locator. However, the presence of polyphenol aids fast and extensive metabolism making its bioavailability significantly lower *in vivo*. To overcome this limitation, Res was successfully loaded on AuNPs, and the resulting Res-AuNP exhibited excellent *in vitro* stability for a period of 48 h and 720 h at 37 °C and 48 °C, respectively. Moreover, at a pH of 7, drug release in PBS was found to be abrupt for the initial 1 h. Afterward, Res-AuNPs were successfully labeled with ^99m^Tc with greater than 95% labeling efficiency with enhanced shelf life. For HT29 cells, both ^99m^Tc-Res-AuNP and ^99m^Tc-AuNP systems showed cell viability above 70%, while cellular uptake was significantly higher for ^99m^Tc-Res-AuNP during the initial 3 h and was retained afterward, as demonstrated by boosted *in vivo* targetability and major accumulation in liver, spleen, and kidney. Thus, for ^99m^Tc-Res-AuNPs, high active uptake and enhanced retention ability in cancer cells made them suitable for *in vivo* cancer imaging [41]. Hepatic carcinoma, which typically develops after chronic liver disease, is the fifth most common cancer type and the third greatest major death cause worldwide [42]. Therefore, developing an NP system with multiple imaging elements and targeting groups is crucial to achieving precise and accurate imaging. One approach is to employ a PEI that shows excellent water solubility, due to various amine groups, which can be utilized for drug loading or coating NPs. A PEI surface was modified with DTPA, PEG, and RGD(RGD-Au PENPs), followed by entrapping AuNPs and ^99m^Tc labeling to develop RGD-^99m^Tc Au PENPs with 99% radiochemical purity [43]. RGD-Au PENPs exhibited higher a CT value, cytocompatibility (for CCK-8 assay), and presence of RGD-developed specific targeting of overexpressed α_v_β_3_ integrin *in vitro*, featuring enhanced cellular uptake with major accumulation in the liver and spleen. SPECT imaging with the RGD-^99m^Tc Au PENPs reflected an intense signal of hepatic carcinoma compared to the unlabeled counterpart that was in good agreement with CT profile, thus making the designed system a potential candidate for SPECT/CT dual imaging. Although SPECT/CT integration is a well-known strategy for tumor imaging and anatomical monitoring, one a recently explored approach is the development of a suitable radiolabeled dendrimer-based NP system with several advantages, such as exceptionally high circulation time in blood, and controlled *in vivo* behavior.

**Figure 6:**
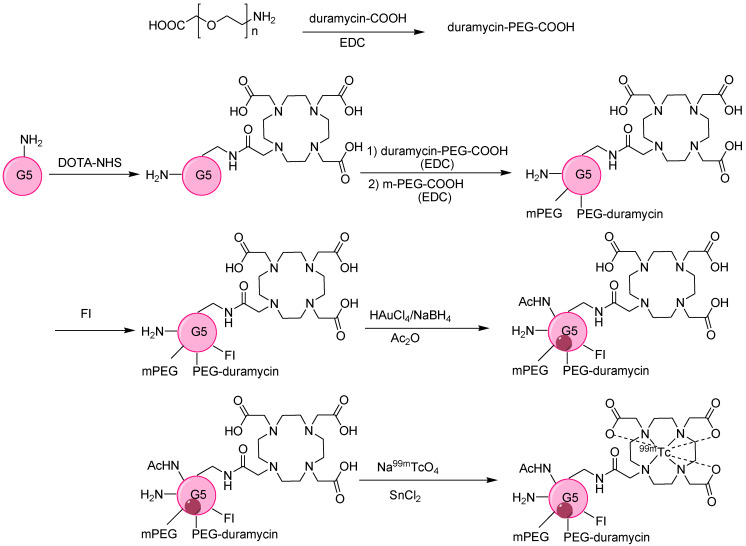
Synthesis of multifunctional {(Au^0^)_200_-G5.NHAc-^99m^Tc-DOTA-FI-mPEG-(PEG-duramycin)} DENPs [44].

Furthermore, the surface functionalization of a dendrimer-based AuNPs (Au DENPs) system with duramycin leads to a biodistribution profile. Hence, after chemotherapy, successful monitoring of tumor apoptosis can continue. The surface of G5.NH_2_ was successfully modified with DOTA, PEG-duramycin, modified mPEG, and fluorescein isothiocyanate (FI) to develop the template of multifunctional dendrimers that serve to entrap AuNPs radiolabeled with ^99m^Tc (Figure 6). The DOTA chelator provided high radiochemical stability above 99%. A comparative analysis of Au DENPs and duramycin Au DENPs in C6 cells confirmed higher fluorescence intensity and twice the contrast enhancement with duramycin Au DENPs [44]. After radiolabeling, ^99m^Tc-duramycin Au DENPs revealed similar trends, with 5.8 times more intense and brighter SPECT images than ^99m^Tc-Au DENPs. *In vivo* studies also confirm the high uptake of ^99m^Tc-duramycin Au DENPs, with major accumulation in the liver and spleen without damaging any organ, thus confirming the developed system was biofriendly and useful for the early monitoring of apoptosis.

**Figure 7:**
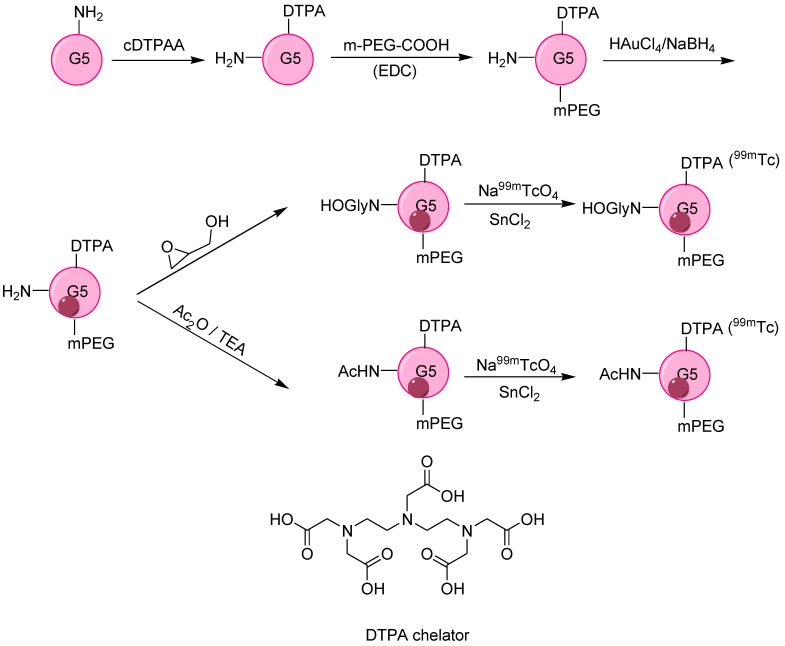
Schematic representation of the synthesis of ^99m^Tc-Au–Gly DENPs and ^99m^Tc-Au–Ac DENPs [46].

Imaging accuracy and sensitivity are greatly dependent on NPs uptake by tumors, and the most common approach in this regard is the surface modification of NPs to develop significant electrostatic interaction between NPs and cell membranes. Following this critical point, a polyethyleneimine (PEI) surface was successfully and sequentially modified by mPEG-COOH, MAL-PEG-SVA (MAL = maleimide, SVA = succinimidylvalerate), DTPA, FI (fluorescein isothiocyanate) followed by entrapping AuNPs, conjugation with alkoxyphenylacylsulfonamide (APAS), and acetylation to yield APAS-Au PENs NPs that were stable, cytocompatible up to 50 °C for 7 days and showed more cellular internalization. Finally, ^99m^Tc labeling was performed to obtain APAS-^99m^Tc-AuPENs with radiochemical purity above 95%. PEI led to the successful entrapping of AuNPs, while APAS modification developed pH-responsive charge transformation (neutral charge without APAS) [45] in the resulting NPs that increased cellular uptake (a pH of 5.5), leading to enhanced SPECT/CT imaging. Moreover, for the same radionuclide dose, SPECT intensity was stronger for APAS-^99m^Tc-Au PENs than for APAS-Au PENs. A novel strategy for SPECT/CT imaging was developed by Wen et al., employing poly(amidoamine) dendrimer (a generation 5 dendrimer, G5-NH_2_) as a template for the synthesis of AuNPs. To obtain the desired biodistribution profile, the amine group of dendrimers was functionalized with hydroxyl (–OH) and acetyl group (–Ac) via hydroxylation and acetylation followed by surface modification dendrimer. Similarly, the DTPA chelator was used to obtain ^99m^Tc-Au-Gly DENPs and ^99m^Tc-Au-Ac DENPs, respectively (Figure 7). ^99m^Tc-Au DENPs exhibit colloidal stability, radio stability, cytocompatibility, and non-toxicity. The –OH or –Ac functionalization led to different aggregation in aqueous solution, as indicated by their SPR peaks at 510 nm and 530 nm, respectively. Moreover, different surface functionalities also led to a unique *in vivo* profile, with major accumulation of both –OH and –Ac-functionalized NPs in the liver accompanied by major detection of ^99m^Tc-Au-Ac DENPs in the lungs and ^99m^Tc-Au-Gly DENPs in blood. The diffusion rate of ^99m^Tc-Au-Gly DENPs was greater than that of ^99m^Tc-Au-Ac DENPs, which was attributed to the lesser hydrodynamic size of the ^99m^Tc-Au-Gly DENPs. However, HU values indicated a balancing the diffusion rate of both DENPs after 4 h post-injection, and both Au DENPs took 168 h to be cleared from the body. The developed ^99m^Tc-labeled Au DENPs with various surface functionalities are considered to be a potential contrast agent for LSN SPECT/CT imaging [46].

### 4.3. Silica Nanoparticles

**Figure 8:**
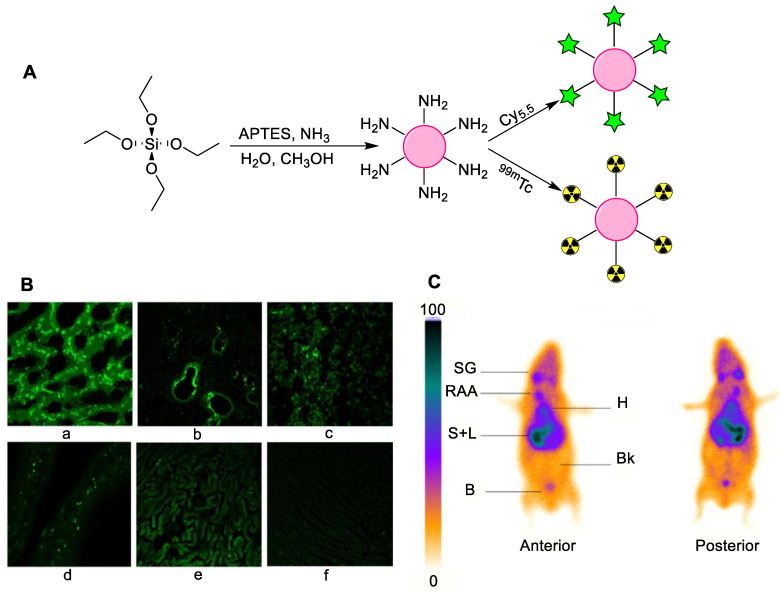
(**A**) Schematic diagram for the synthesis of Cy5.5 or ^99m^Tc conjugated silica nanoparticles; (**B**) biodistribution of Cy5.5-conjugated silica nanoparticles in (**a**) liver (**b**) lung (**c**) spleen (**d**) testis (**e**) kidney and (**f**) heart, 2 h post-oral administration; (**C**) scintigraphic images of ^99m^Tc radiolabeled silica NPs after oral administration (SG—salivary glands, RAA—radiotracer administration aria, H—heart, S—spleen, L—liver, B—bladder, Bk—background). Reprinted with permission from [49], with permission from Elsevier, 2015.

Silica NPs, particularly mesoporous silica NPs, have shown their potential as drug carriers. Their unique structure, chemical stability, biocompatibility, and surface functionalization enable the delivery and the controlled release of various pharmaceuticals. Mesoporous silica NPs have a rigid structure with porous morphology and large surface area, enabling the attachment of various drugs, tumor targeting peptides and proteins, functional groups, chelators, and radioisotopes [47]. Soon after their discovery, mesoporous silica NPs were modified with a variety of drugs and radioisotopes, particularly the most widely used ^99m^Tc. In the first example, mesoporous silica SBA-15 was prepared and radiolabeled by employing SnCl_2_ and mesoporous silica in a reaction for 20 min, followed by 10 min incubation with ^99m^Tc. The direct radiolabeling provided a highly stable radiotracer with more than 99% labeling stability, as tested over 8 h. Preliminary cytotoxicity results (without ^99m^Tc radiolabeling) verified non-cytotoxicity. The results showed that aptamer-loaded mesoporous silica NPs are of great practical interest and can be a potential candidate in nuclear medicine [48]. For further investigation, silica NPs were conjugated with Cy5.5 or ^99m^Tc, followed by characterization and evaluation *in vitro* and *in vivo* by scintigraphic imaging. Furthermore, biodistribution studies were performed using intravenous injection or oral administration. The surfaces of as-prepared silica NPs were functionalized with an amine moiety having Cy5.5 fluorescent and ^99m^Tc radiolabeling possibility (Figure 8). The particle size distribution was around 200–350 nm, while the surface charge was modified from negative to positive after amino functionalization. The NPs’ enhanced uptake was observed in kidneys, lungs, liver, spleen, and testis for the fluorescent silica. For the radiolabeled silica NPs, the toxicity level was significantly reduced as shown by the biodistribution profile, thus making radiolabeled silica NPs possible candidates for employment in nuclear medicine as a radiotracer [49].

The direct radiolabeling of silica NPs provided satisfactory results regarding *in vitro* and *in vivo* stability. However, chelator-based radiolabeling techniques still provide superior results. After synthesis, radiolabeled mesoporous silica NPs (MSNs) were allowed to functionalize with APTES, followed by anchoring through DTPA as a ^99m^Tc chelator. The product was purified by ultracentrifugation to obtain high radiochemical yields (over 95%) and enhanced stability [50]. For ^99m^Tc-DTPA-MSN, high uptake in the liver was observed, with fast clearance from the blood, followed by a maximum accumulation of tracer in the lungs, thus making these structures a potential candidate for theranostic purposes. The radiolabeling and biodistribution studies using the silica NP platform demonstrated promising results, and stimulated further development. HER2 is found to be overexpressed in breast cells, and modified silica NPs were synthesized to target HER2. For this, PAMAM was embedded on the exterior of amorphous silica NPs, with a grafting percentage of 55.6%. Moreover, processed NPs (PCSNs) were conjugated to indocyanine green (ICG), a known NIR (Near-infrared) fluorescent dye, by incubation at 37 °C for 30 min. ^99m^Tc was attached to the PAMAM amine surface via direct chelation to synthesize the dual-imaging probe. Additionally, for better comparison, two chelating agents, DTPA and mercaptoacetyltriglycine (MAG_3_), were employed. Anti-HER2 antibody was attached to the surface of the PCSNs to increase specificity towards HER2 receptors. The HER2 positive cells exhibited stronger NIR fluorescence compared to HER2 negative cells. The chelator-based radiolabeling showed high *in vivo* stability as compared with direct radiolabeling using PAMAM. Interestingly, the DTPA chelator showed a high radiolabeling yield as compared with MAG3 [51]. Recombinant humanized monoclonal antibodies, such as Trastuzumab (TZ), can recognize the extracellular domain of HER2 protein and are considered to be among the best cancer therapeutic drugs. Several imaging agents were conjugated with TZ to target HER2-positive breast cancer. Recently, Belloliet et al. explored TZ conjugated silica NPs to target HER2-positive breast cancer. For the radiolabeling of SiNPs, firstly (^99m^Tc[CO]_3_)^+^ was prepared, followed by (^99m^Tc[CO]_3_)^+^—His-Tag prelabeling. Then, the coupling of (^99m^Tc[CO]_3_)^+^—His-Tag with SiNP-NTA was achieved. Finally, (^99m^Tc[CO]_3_)^+^—His-Tag -SiNP-NTA were conjugated with TZ. Afterward, ^99m^Tc radiolabeled nanoparticles with or without TZ were tested for breast cancer imaging. Biodistribution studies for HER2+ cells confirmed that, for ^99m^Tc, the nanosilica uptake was higher than was evident from *in vitro* results. Moreover, in all cell cultures, toxicity tests confirmed the NPs’ safety. Hence, Belloliet et al. emphasized further evaluating ^99m^Tc-nanoconjugates with silica to introduce safe and multipurpose nano-receptors to analyze and treat aggressive breast cancer [52].

In an attempt to develop anti-HER2 antibodies, trastuzumab (TZ) is recently employed composition. However, toxicity and development of resistance in a patient are the drawbacks associated with this approach. To overcome these limitations, TZ was installed on radiolabeled silica NPs (^99m^Tc-SiNPs-TZ) for SPECT imaging. An optimized ratio of silica NPs to TZ was found to be 1:2 and 1:8. Higher accumulation was found for SiNPs-TZ with low toxicity; meanwhile, for better comparison, the DOX (doxorubicin)-loaded system DOX-SiNPs-TZ was studied and a similar trend was found. However, the reduction in tumor volume by DOX-SiNPs-TZ (1:8) was more significant than that of DOX-SiNPs, thus signaling its potential to be used as an innovative system for treating HER2-positive breast cancer [53]. ^99m^Tc radiolabeled silica NP based radiotracers have not only tested for HER2 positive cancer cells, but efforts have also been made to target other cancer types, such as melanoma. Melanoma is considered to be among the most dangerous cancers, and it cannot be detected and treated early. So, there was a need to develop a unique drug delivery system that could detect such tumors. One approach in this regard was utilizing mesoporous silica NPs with magnetic cores followed by dacarbazine doping and ^99m^Tc labeling supporting subsequent employment as an imaging agent for SPECT. Labeling efficiency was found to be greater than 98%, and the resulting particles exhibited non-cytotoxicity and stability for a period of 8 h, entrapment efficiency of more than 98%, and enhanced uptake in the spleen and liver.

**Figure 9:**
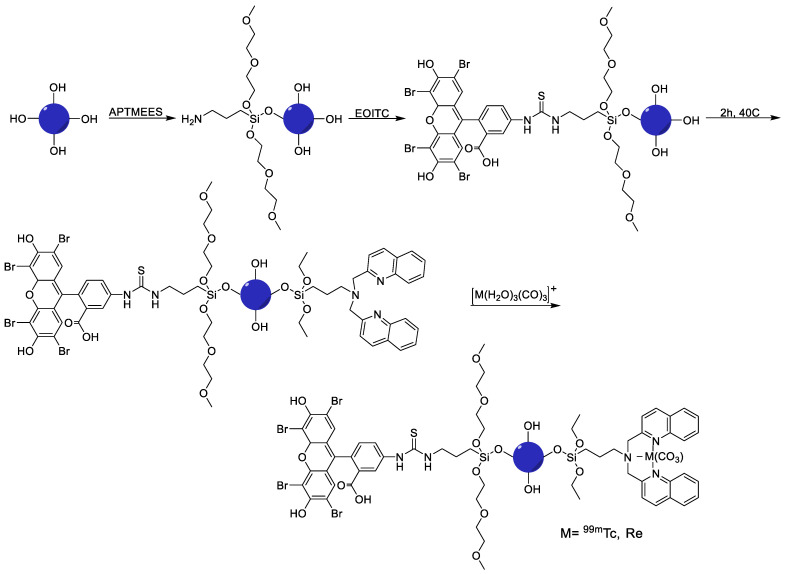
Synthesis of multifunctional mesoporous silica nanoparticles (SBA-15). Step 1: installation of APTMEES at room temperature, Step 2: conjugation of eosin isothiocyanate (EOITC) fluorescent dye, Step 3: conjugation of ^99m^Tc/^188^Re chelator, Step 4: radiolabeling with ^99m^Tc or ^188^Re [55].

Furthermore, the EPR effect of synthesized and labeled NPs was confirmed by *in vivo* studies. However, targeting time was significantly longer, but the drug adopts two ways to reach the tumor: intratumorally, and by a systemic mode that makes this developed system a reliable and efficient nano-imaging agent for melanoma [54]. Therefore, the silica NP platform was tested for optical and SPECT imaging and also considered for development as a radiotherapy platform. For this, taking advantage of photophysical characteristics of the [M(CO)_3_]^+^ fragment where M = Re and the radiation characteristics of ^186/188^Re and ^99m^Tc, a multi-model silica platform was developed with ^99m^Tc/Re as a theranostic pair with high potential for nanomedical applications. Furthermore, to synthesize mesoporous silica NPs, the most suitable mesoporous silica construct was screened, suitable chelate systems were employed and particle surfaces was modified to achieve the required targeting efficiency with no change in the bio profile of the silica platform. Thus, synthetic protocols were successfully employed to develop a target-specific theranostic nano platform by enabling a blend of fluorescence (eosin isothiocyanate (EOITC)) and radio imaging (Figure 9), hence paving the route for chemo and radiotherapy along with SPECT and optical imaging [55].

**Figure 10:**
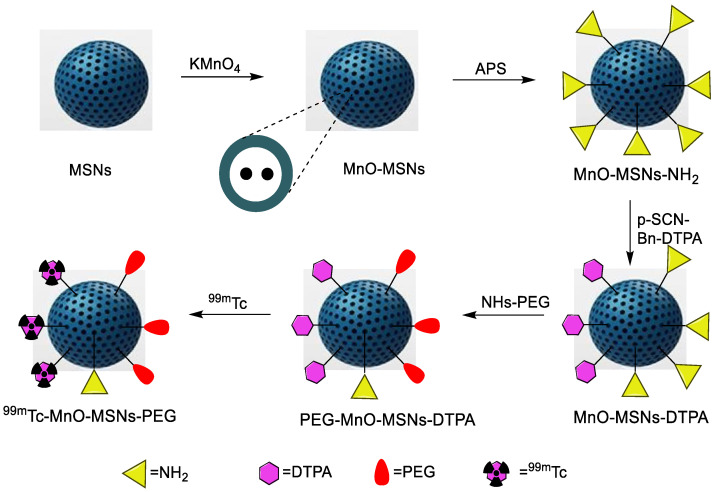
Schematic diagram for the synthesis of the ^99m^Tc-MnO-MSNs-PEG nanodrug [56].

Silica NPs have demonstrated excellent results when combined with cancer-targeting biomolecules, theranostic radioisotopes, fluorescent imaging agents, and chemotherapeutic drugs. Recently, silica NPs were employed for a dual probe to merge their features of extraordinary SPECT sensitivity and enhanced MRI resolution. Since manganese-based T_1_ contrast agents showed lower toxicity than gadolinium-based contrast agents. Here, MnONPs were synthesized and dispersed inside mesoporous silica NPs (MnOx-MSNs), followed by ^99m^Tc, to be employed for SPECT-MRI dual-modal imaging. Furthermore, DOX was loaded to explore chemotherapeutic efficiency. A novel ^99m^Tc-Mn-MSNs-PEG material was thus developed (Figure 10) for evaluating pH-responsive MRI and SPECT. The radiolabeling yield was above 99% and, owing to the pH-dependent nature of MnOx-MSNs, the r_1_ value for nanoprobe reached 6.60 mM^−1^s^−1^. Moreover, this nanostructure showed the potential to simultaneously deliver drugs and release them at the tumor location, making it an ideal platform for biological imaging and chemotherapy [56]. A SPECT imaging and biodistribution study confirmed high uptake in the tumor, liver, and lungs.

### 4.4. Titanium Nanoparticles

Titanium dioxide is a common material which has various applications in consumer products. Chemical and radiation stability, low toxicity, and biocompatibility are attractive features that encourage its use in medicine and pharmacy. Additionally, its synthesis is simple and fast, and large-scale production is possible. In nuclear medicine, titanium oxide is used as an adsorbent in a ^68^Ge/^68^Ga generator. In the past, TiO_2_ was radiolabeled with various radioisotopes, such as vanadium-48, flourine-18, and astatine-211 for pharmacokinetic studies. Recently TiO_2_ NPs were radiolabeled with radium-223 to explore its therapeutic potential of the radiotracer.

Moreover, the NPs were also radiolabeled with ^99m^Tc to investigate *in vivo* biodistribution [57]. The TiO_2_NPs were radiolabeled with ^99m^Tc using surface adsorption and intrinsic radiolabeling techniques. Both strategies showed high radiolabeling efficiency. However, in the *in vitro* experiments, low radiolabeling stabilities were observed in various media. Therefore, it is expected that surface modification and chelator-based radiolabeling can provide better results.

**Table 2:** Summary of ^99m^Tc labeled inorganic nanoparticles and their applications.

Nanoparticle	Application	Drug Loaded on NPs	Radiolabeling Method	Ref.
Iron oxide Nanoparticles	Hyperthermia procedure	Without drug	Direct radiolabeling using tetrahydroborate exchange resin (reducing agent)	[27]
	SPECT and MRI based imaging of hepatocytes	Lactobionic acid	Chelator-based radiolabeling using DTPA and stannous chloride (reducing agent)	[28]
	The liver and spleen imaging	Without drug	Chelator-based radiolabeling using bisphosphonate chelator	[29]
	The liver and spleen imaging	Dimerccaptosuccinic acid (DMSA)	Chelator-based radiolabeling using DMSA	[30]
	SPECT and MRI imaging of H1299 α_v_β_3_-positive cells	c(RGDyC) peptide	Chelator-based radiolabeling using DTPA and stannous chloride (reducing agent)	[31]
	α_v_β_3_-positive tumor imaging	c(RGDyC) and glutathione (GSH)	Direct radiolabeling using stannous chloride (reducing agent)	[32]
	Fe_3_O_4_ and CoFe_2_O_4_ nanoparticles for liver and spleen imaging	Without drug	Direct radiolabeling using stannous chloride (reducing agent)	[33]
Gold Nanoparticles	GRP-r receptor-based therapy of prostate cancer	Lys^3^-bombesin and HYNIC-Gly-Gly-Cys-NH_2_ [HYNIC (hydrazinonicotinamide), GGC(Gly-Gly-Cys) peptide	Chelator-based radiolabeling using HYNIC and stannous chloride (reducing agent)	[35]
	Lymph node (SLN) imaging	Without drug	Chelator-based radiolabeling using HYNIC and stannous chloride (reducing agent)	[36]
	α_v_β_3_-positive tumor imaging	HYNIC-GGC and cyclic[Arg-Gly-Asp-Phe-Lys(Cys)] {c[RGDfK(C)]}.	Chelator-based radiolabeling using HYNIC and stannous chloride (reducing agent)	[37]
	GRP-r-positive tumors, SLN detection and α_v_β_3_ positive tumors	Lys^3^-bombesin, thiol-mannose or cyclo[Arg–Gly–Asp–D–Phe–Lys–(Cys)] c[RDGfK(C)]	Chelator-based radiolabeling using HYNIC and stannous chloride (reducing agent)	[38]
	Plasmonic photothermal therapy	HIV Tat (49–57) peptide and bombesin	Chelator-based radiolabeling using HYNIC and stannous chloride (reducing agent)	[39]
	SPECT/CT imaging of tumor xenografted model	Poly(amidoamine) (PAMAM) dendrimers	Chelator-based radiolabeling using DTPA and stannous chloride (reducing agent)	[40]
	HT29 cells	Resveratrol (Res)	Direct radiolabeling using stannous chloride (reducing agent)	[41]
	α_v_β_3_-positive tumor imaging	c(RGDyC) peptide	Chelator-based radiolabeling using DTPA and stannous chloride (reducing agent)	[43]
	Monitoring of tumor apoptosis	Duramycin	Chelator-based radiolabeling using DOTA and stannous chloride (reducing agent)	[44]
	Tumor targeting	Alkoxyphenylacylsulfonamide (APAS)	Chelator-based radiolabeling using DTPA and stannous chloride (reducing agent)	[45]
	Tumor targeting	Generation 5 dendrimer, G5-NH_2_	Chelator-based radiolabeling using DTPA and stannous chloride (reducing agent)	[46]
Silica Nanoparticles	General biodistribution study	Without drug	Direct radiolabeling using stannous chloride (reducing agent)	[48]
	General biodistribution study	Without drug and with Cy5.5 fluorescent agent	Direct radiolabeling using stannous chloride (reducing agent)	[49]
	General biodistribution study	APTES modified nanoparticles	Chelator-based radiolabeling using DTPA and stannous chloride (reducing agent)	[50]
	HER2 receptors targeting in tumors	Anti-HER2 antibody	Direct radiolabeling using stannous chloride (reducing agent),Chelator-based radiolabeling using DTPA and MAG3	[51]
	To target HER2 positive breast cancer	Trastuzumab (TZ)	Chelator-based radiolabeling using His-Tag	[52]
	To target HER2 positive breast cancer	Trastuzumab (TZ) and DOX	Direct radiolabeling	[53]
	Melanoma treatment	Dacarbazine	Direct radiolabeling using stannous chloride (reducing agent)	[54]
	General biodistribution study	Without the drug, radiotherapy using ^186/188^Re and optical imaging using eosin isothiocyanate (EOITC)	Direct radiolabeling using a tricarbonyl kit	[55]
	General biodistribution study and dual SPECT and MRI imaging agent	DOX drug for chemotherapy and MnO for MRI imaging	Direct radiolabeling using stannous chloride (reducing agent)	[56]
Titanium Nanoparticles	General biodistribution study	Without drug	Direct radiolabeling on the surface of nanoparticles	[57]

## 5. Organic Nanoparticles

Over the last decade, various organic NPs such as proteins, liposomes, polymeric micelles, and dendrimers have been synthesized and used in various applications of the life sciences. Moreover, organic NPs radiolabeled with various radioisotopes have been studied for molecular imaging and radiotherapy applications. The key ^99m^Tc radiolabeled organic nanoparticles are summarized in Table 3.

### 5.1. Dendrimers

Dendrimers are homogeneous, highly symmetric, branched polymeric molecules with several applications in the biomedical and pharmaceutical industry. Dendrimers can be synthesized using various chemical compositions in different molecular weights and sizes. Moreover, radioisotopes and drugs can be loaded on the dendrimer surfaces or encapsulated inside them to synthesize multipurpose imaging and therapeutic agents. Recently, dendrimers have been employed to target the folate receptor, which is overexpressed in various human diseases such as lung, kidney, ovary, and breast cancers. The folate receptor has a high affinity towards folic acid moieties, so positively charged PAMAM G4 dendrimers were decorated with folic acid. The surface modification was done using a PEG polymer to stabilize NPs, restrict serum protein binding, and increase biocompatibility. ^99m^Tc radiolabeling was performed using a HYNIC chelator. The radiolabeled nanomaterial was obtained in high radiochemical yield and purity. The radiotracer showed excellent *in vitro* stability in mouse serum and PBS buffer solution. Moreover, high cellular uptake was observed in the human breast cancer (MCF-7) cell line, as compared to normal cells. The gamma camera images were obtained using breast cancer-bearing female BALB/c mice. The gamma camera images and biodistribution studies showed high uptake in the liver and spleen. However, only a moderate uptake was observed in the tumor [58]. In another study, the negatively charged dendrimer-G2 was explored. For this, citric acid and polyethylene glycol (PEG) was used to synthesize negatively charged dendrimers. This approach circumvented the need for a chelator for ^99m^Tc radiolabeling.

Moreover, these negatively charged dendrimers protect against the toxic interaction between the tracer and the healthy cells. Glutamine amino acid, which plays an essential role in metabolic pathways, was installed on these dendrimers to detect cancer cells *in vivo*. Direct ^99m^Tc radiolabeling was performed using tin chloride and ascorbic acid. The radiolabeling yield was more than 94%, and the radiotracer showed high *in vitro* stability in PBS and human serum. The biodistribution study and gamma camera images showed high radioactivity accumulation in the kidneys because of the lipophilicity of the radiotracer.

Additionally, the low-level signal in the stomach and thyroid confirmed the *in vivo* stability of the product. Finally, moderate uptake was observed in the A549 tumor 2 h post intravenous injection. These results promised further studies at the preclinical and clinical levels [59].

### 5.2. Polymeric Nanoparticles

**Figure 11:**
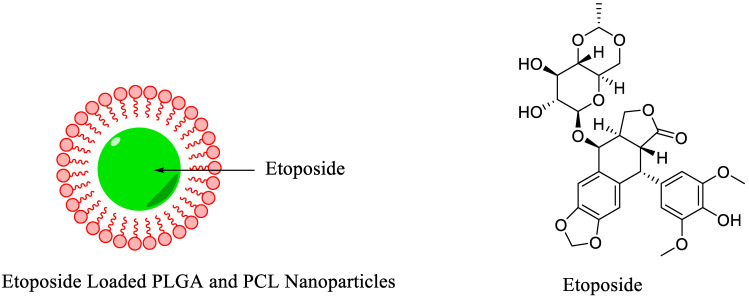
Schematic diagram of Etoposide-loaded PLGA and PCL nanoparticles and chemical structure of Etoposide [60].

Currently, various non-toxic and biodegradable polymers are being used to synthesize polymeric NPs in the form of nanospheres and nanocapsules. These polymeric NPs have been extensively used in biomedical applications because of their outstanding characteristics such as simple design and synthesis, biocompatibility, facile surface modification, and wide structural varieties. The polymeric NPs showed promising results in preclinical studies, such as the controlled release of the drug, targeted drug delivery for cancer treatment, gene therapy, and multimodality molecular imaging. Recently, biodegradable, biocompatible, FDA-approved polymers such as poly (lactic-co-glycolic) acid (PLGA) and poly-ε-caprolactone (PCL) were used to synthesize polymeric NPs. PLGA and PCL NPs demonstrated promising results in the controlled release of the drugs. However, the *in vivo* imaging, biodistribution, and pharmacokinetic studies provided crucial data regarding the delivery of the administrated cancer drugs and the efficiency of the ongoing treatment procedure. For this, a study was conducted in which a cancer drug, Etoposide, was loaded on PLGA and PCL NPs (Figure 11). The biodistribution and pharmacokinetic data were obtained using a ^99m^Tc radioisotope. Etoposide-loaded polymeric NPs were radiolabeled using a direct radiolabeling strategy where stannous chloride was used as a reducing agent. The radiotracers showed moderate stability in saline and rabbit serum for more than 24 h. Radiolabeled NPs showed high retention in the blood and liver as compared with radiolabeled Etoposide. Overall, polymeric NPs significantly increase the biological half-life of the drug [60].

Radiolabeled polymeric NPs were also used as an alternative to classical radiopharmaceuticals. For instance, ^99m^Tc labeled human serum albumin and sulfur nano-colloid have been used for sentinel lymph node imaging. Both radiopharmaceuticals have few intrinsic limitations. ^99m^Tc PLGA NPs were synthesized as an alternative radiotracer for sentinel lymph node detection. PLGA NPs were radiolabeled with ^99m^Tc using a chelator-free approach [61]. The radiolabeling yield was low, and additional purification of the radiotracer was required to achieve high specific activity. However, the biodistribution and scintigraphic imaging showed promising results. High radioactivity was observed in the sentinel lymph node. In another study, efforts were made to improve radiolabeling yield and stability. The carboxylic acid groups on the surface of PLGA NPs were modified with *p*-aminobenzyldiethlenetriaminepentaacetic acid (*p*-NH_2_-Bz-DTPA). The DTPA chelator was used to increase the radiochemical yield and stability of the complex.

Interestingly, a highly stable complex was obtained under moderate reaction conditions. A biodistribution study on Wistar rats showed increased uptake in the sentinel lymph nodes. The studies confirmed that chelator-based radiolabeling provides better results in terms of stability and biodistribution. Long blood circulation and low liver uptake were observed after intravenous injection for fluorescent dye or ^131^I-labeled PLGA NPs. However, stannous chloride-catalyzed ^99m^Tc-radiolabeled PLGA NPs showed contrary results, such as high liver uptake and short blood life. Therefore, it was assumed that the production ^99m^Tc stannic oxide colloid during the radiolabeling of PLGA NPs was influencing the biodistribution results. PLGANPs were radiolabeled using stannous chloride, sodium dithionite, or sodium borohydride as reducing agents to confirm the hypothesis. It was observed that stannous chloride-mediated ^99m^Tc-labeled PLGA NPs showed high liver uptake because of the formation of a ^99m^Tc stannic oxide colloid. Sodium borohydride provided a volatile radiolabeled complex.

Sodium dithionite produced highly stable radiolabel NPs without the formation of a ^99m^Tc colloid. Moreover, biodistribution studies confirmed low liver uptake and high blood circulation time [62]. The literature survey reveals that only a handful of polymeric NPs were radiolabeled with ^99m^Tc and chitosan-based polymeric NPs. The chitosan polymer demonstrated remarkable properties such as an antibacterial property, low immunogenicity, biodegradability, biocompatibility, and non-toxicity; therefore, this material has found wide applications in various areas, such as food additives, pharmaceuticals, and biomedicine. Recently, poly-gamma glutamic biopolymers and chitosan were used to synthesize polymeric NPs. These polymeric NPs were further decorated with folic acid to target folate receptor-overexpressed tumor cells. The NPs were radiolabeled using a ^99m^Tc radioisotope and stannous chloride as reducing agents. The radiotracer showed high *in vitro* and *in vivo* stability. Moreover, high radioactivity was observed in the liver and in tumor-bearing kidneys. The biodistribution studies and SPECT/CT images were compatible; high uptake was observed in folate receptor-overexpressing tumors [63].

There are only a few examples in the literature where polymeric NPs were modified to target more than one receptor. In an example, PLGA NPs were modified with folic acid (FA) to target folate receptors, and with K237 (HTMYYHHYQHHL) peptide to target vascular endothelial growth factor receptor-2 (VEGFR-2). The NPs were further modified with polyethylene glycol (PEG) as an outer corona to enhance blood circulation time and reduce nonspecific interface with blood components. These K237/FA-PEG-PLGA NPs were radiolabeled with ^99m^Tc using a direct radiolabeling strategy. The radiotracer showed high *in vitro* stability in saline and serum, and more than 90% of the radioactivity remained intact after 24 h. The radiolabeled NPs showed high binding specificity to SKOV-3 cell lines. A scintigraphic imaging and biodistribution study was performed in xenografted mice (SKOV-3 cells) using an intravenous injection. High radioactivity was observed in the tumors, liver, kidneys, and blood [64]. The high activity in the blood revealed a prolonged circulation time. Apart from receptor targeting, some chemotherapeutic drugs were also loaded on polymeric NPs to increase biological half-life, control drug release, or increase solubility.

In another example, biocompatible and biodegradable polymers, polylactic acid (PLA), and polyethylene glycol (PEG) were synthesized into polymeric PLA-PEG polymeric NPs. These NPs were loaded with Gemcitabine, a common chemotherapeutic drug used in pancreatic, bladder, lung, ovarian, and breast cancer. Finally, Gemcitabine-loaded PLA-PEG polymeric NPs were radiolabeled with ^99m^Tc to monitor biodistribution and pharmacokinetics in normal mice. The radiolabeling of NPs was performed using a tricarbonyl kit ([^99m^Tc (CO)_3_(H_2_O)_3_]^+^). The radiolabeled complex showed high *in vitro* stability in saline. The radiotracer was intravenously injected into normal rats. A uniform distribution of the radiotracer was observed in the body. As shown in SPECT images, high uptake was observed in the liver, lungs, and kidneys [65].

### 5.3. Lipid Nanoparticles

Lipids are hydrophobic biomolecules that include glycerides, fatty acids, phospholipids, sphingolipids, sterols, and prenol (based on their chemically functional backbone). Lipids play a vital role in energy metabolism and storage, signaling, and hormones [66]. Major advantages in drug development associated with lipids are their high biocompatibility and biodegradability, along with their ability to serve as efficient transporters for hydrophobic drugs. Depending upon their structure, lipid-based NPs can be categorized into liquid NPs, solid lipid NPs, lipid nanoemulsions, and nanostructured lipid carriers [67]. For cisplatin, hybrid nucleoside lipids (NLs) allow well-organized encapsulation, so advanced formulations for PEG NLs were prepared, followed by ^99m^Tc radiolabeling without chelators. The formulation of these NPs involved surface modification of the PEG with targeting agents. Therefore, to achieve active targeting by FA functionalization, uridine was firstly pegylated to aid ^99m^Tc radiolabeling. The labeling efficiency was found to be more than 97%. Thus, the resulting functionalized and labeled NPs (^99m^Tc–NP-PEGFA) enhanced the half-life of cisplatin *in vivo* with major accumulation in tumor tissue, improved the pharmacokinetic profile, and had rapid clearance. *In vitro*, these hybrid NPs were stable, and actively internalized two types of cell lines with overexpressed folic acid receptors, thus making a significant contribution to efficient and non-toxic drug delivery systems [68]. Among well-known radiopharmaceutical and bone imaging agents, ^99m^Tc-MDP (^99m^Tc labeled methylene diphosphonate) is considered one potential candidate as it accumulates in bone and possesses an easily recognizable biodistribution profile. Radiolabeled ^99m^Tc-MDP-encapsulated solid lipid NPs (SLNs) less than 500 nm in size were designed that successfully encapsulate the ^99m^Tc radiotracer. With both administration modes adapted for ^99m^Tc-MDP-SLNs, SPECT/CT imaging further confirmed biodistribution and high uptake in the bones, with major accumulation in the liver, spleen, and stomach. ^99m^Tc-MDP oral administration did not show any uptake. However, the use of SLNs still needs to be optimized to avoid non-target uptake and to allow the successful oral administration of ^99m^Tc based radiopharmaceuticals relative to the traditional i.v. administration mode [69].

### 5.4. Liposomes

**Figure 12:**
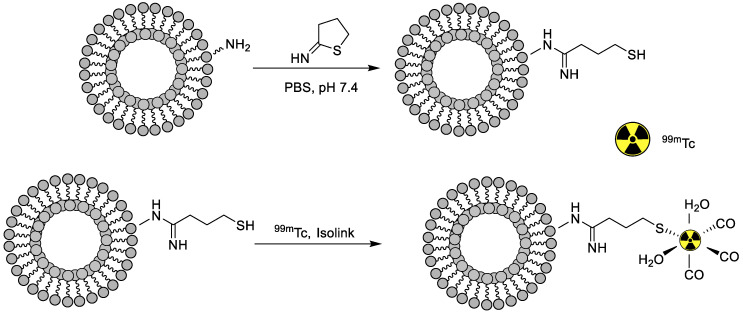
The surface modification of liposomes using 2-iminothiolane and ^99m^Tc radiolabeling [72].

A liposome is a small, bio-compatible spherical-shaped vesicle composed of cholesterol and non-toxic phospholipids that can encapsulate hydrophilic or lipophilic drugs. Their properties can be engineered considerably, by varying their composition, size, surface charge, and method of preparation. Moreover, rigidity or fluidity is determined by choice of the bilayer components and the charge of the bilayer [70]. Liposomes are broadly classified as: (i) unilamellar vesicles (UVs) (composed of one phospholipid bilayer), and (ii) multilamellar vesicles (exhibit multiple phospholipid bilayers). UV’s can be further subcategorized as: (i) large UVs and (ii) small UVs. When the liposomes are functionalized with PEG, they give steric hindrance to the phagocyte system and prolonged blood circulation time. Moreover, the liposomes’ PEGylation reduces radiolabeling efficiency (RE) when the glutathione method is employed for radiolabeling. Therefore, the effect of the liposomal PEG extent (PEGExt) on the *in vivo* biodistribution profile in Wistar rats and radiolabeling efficiency with ^99m^Tc was investigated. PEGylated liposomes were prepared, followed by ^99m^Tc radiolabeling with a labeling efficiency of more than 90% in all compositions. It was found that PEGylated liposome size was reduced by increasing the PEGExt, and with this increased PEGExt, radiolabeling stability was found to significantly enhanced in human serum, with major accumulation in the liver and the spleen, while, with increasing PEGExt, the accumulation in the organs decreased, and uptake was found to be dependent on the liposomal size. Better and improved circulation time was observed for PEGylated liposomes compared to liposomes without PEG chains [71]. Thus, the liposome designs significantly influenced the *in vivo* biodistribution profile of medicines. Moreover, the radiolabeling of liposomes and their potential application for imaging is a significantly growing area of interest. In this line, a novel approach was developed in which the surfaces of liposomes were labeled with ^99m^Tc by using the iminothiolane- ^99m^Tc-tricarbonyl complex (Figure 12) with 95% labeling efficiency, stability of over 2 h in bovine serum, and a highest reported specific activity of 50 MBq, paving the way for quantitative SPECT imaging with the developed system. Afterward, the resulting system was bound to the surface of the liposome. *In vivo* labeling stability was further confirmed by quantitative SPECT/CT studies showing significant differences between the labeled liposomes and the free ^99m^Tc-tricarbonyl and non-PEGylated liposomes, resulting in fast clearance from blood with major accumulation in the liver and spleen [72].

### 5.5. Oligomers

**Figure 13:**
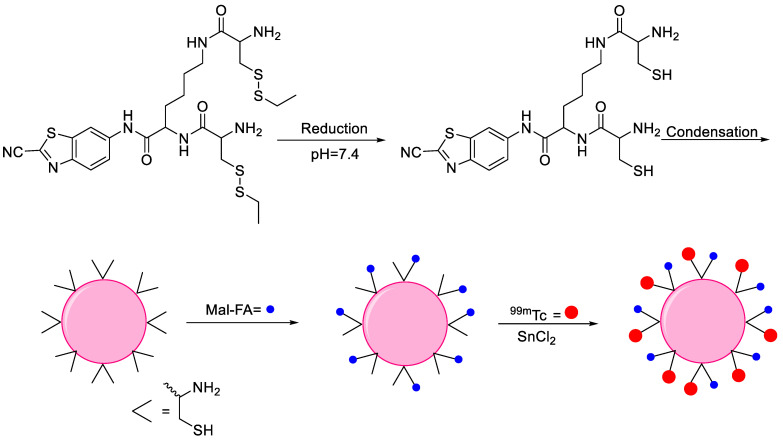
Synthesis of ^99m^Tc radiolabeled oligomer nanoparticles [74].

Oligomers are a class of polymers whose molecular weight is relatively low, owing to the limited number of repeating units, and this number plays a crucial role in determining their physical properties during polymerization reactions. Oligomers typically form as intermediates. Until now, the diagnosis and effective treatment of proteinopathies is in the early stages, and one promising candidate in this regard is an oligomer that offers significant advances in the treatment of these diseases [73]. Only a single example is available in the literature where oligomer NPs were radiolabeled with ^99m^Tc for SPECT imaging. The design and synthesis scheme are noted mentioned in Figure 13. 2-cyanobenzothiazole (CBT) was used to synthesize an intermediate compound CKC-CBT and the tris(2-carboxyethyl)phosphine (TCEP)-based condensation reaction of CKC-CBT provided biocompatible oligomer NPs through the π-π interaction. Maleimide-folic acid (Mal-FA) was employed to develop covalent conjugation between folic acid and the SH of the NPs to target the folate receptor. ^99m^Tc was conjugated with cysteine via amine and the thiol group on the surface of NPs to prepare NPs-FA-^99m^Tc with more than 97% radiolabeling efficiency and pronounced stability without DOTA, owing to the presence of multiple complexation sites on the NP surfaces. The direct radiolabeling reaction using stannous chloride provided a high radiolabeling yield. NPs-FA-^99m^Tc demonstrated high *in vitro* uptake in human HepG2 cells that were folate receptor-positive. The results encouraged further *in vivo* investigation, such as detailed biodistribution and SPECT imaging in animal models [74].

### 5.6. Protein Nanoparticles

Proteins are large, complex molecules involved in most cellular works and are critical for the building, proper functioning, and regulation of the various tissues and organs. The transport of insoluble drugs by using NPs is receiving increaded attention because they dissolve quickly in the bloodstream and reach a specific target due to their small size. Proteins exhibit unique properties and functions in biological materials, making them effective base materials for the production of NPs [75]. Therefore, protein NPs are actively employed as medicinal and functional tools due to their abundance in natural sources, cost-effectiveness, low toxicity, and biodegradability [76]. Among various available proteins, human serum albumin (HSA) protein-based NPs have demonstrated unique features such as high drug loading capacity and easy surface modification using polymers, finding use in cancer-targeting drugs, SPECT, and PET-based radioisotopes, among others. In the first example, HSA NPs loaded with bevacizumab (NP-Ab) were radiolabeled with ^99m^Tc, and their biodistribution and SPECT imaging ability was investigated using rodents. In order to obtain an idea about the biodistribution profile of the individual shell and the cargo of NP-Ab, they were functionalized with PEG35000 and studied by SPECT/CT imaging; thus, two formulations were investigated. In the first approach, a desolvation strategy was employed to synthesize NP-Abs, followed by PEG35000 coating and ^99m^Tc radiolabeling via direct radiolabeling or the tricarbonyl kit methodology ([^99m^Tc] Tc-NP-Ab). In the second approach, Ab radiolabeling via [^99m^Tc] [Tc (CO)_3_(H_2_O)_3_]^+^ was performed that was further employed to synthesize NP-[^99m^Tc]Tc-Ab. The radiochemical purity of both nano drugs was >95% and more than 87% of radioactivity remained intact when incubated in plasma for 24 h. The biodistribution study and SPECT/CT images confirmed high uptake of the [^99m^Tc] Tc-NP-Ab tracer in the liver and kidneys. On the other hand, NP-[^99m^Tc] Tc-Ab mainly accumulated in the liver, and clearance was slow. Thus, the different clearance rates for the antibody and shell provided the desired imaging information for the drug delivery of nano-systems [77]. In an attempt to optimize the radiolabeling of polymer-coated albumin NPs, the desolvation method was employed to synthesize the NPs using albumin, and NOTA-functionalized albumin was coated with four distinct polymers, such as HPMC, GMN2, GPM2, and GTM2 and subsequently radiolabeled with ^99m^Tc and ^67^Ga at different times and temperatures. With both approaches, radiochemical purity was above 97%, and the NPs demonstrated enhanced *in vitro* stability even after 48 h of labeling, i.e., 70% for ^99m^Tc and 90% for the ^67^Ga-based radiolabeled NP system. Thus, the biodistribution profile of the ^99m^Tc -GPM2 and ^67^Ga -NOTA-GPM2-based NPs systems showed major accumulation in the liver 2 and 24 h after i.v. administration [78].

**Table 3:** Summary of ^99m^Tc-labeled organic nanoparticles and their applications.

Nanoparticle	Application	Drug Loaded on NPs	Radiolabeling Method	Ref.
Dendrimer	Human breast cancer (MCF-7) imaging	Without drug	Chelator-based radiolabeling using HYNIC and stannous chloride (reducing agent)	[58]
	A549 tumor imaging	Without drug	Direct radiolabeling using stannous chloride (reducing agent)	[59]
Polymeric Nanoparticles	General biodistribution study	Etoposide	Direct radiolabeling using stannous chloride (reducing agent)	[60]
	Sentinel lymph node	Without drug	Direct radiolabeling using stannous chloride (reducing agent),chelator-based radiolabeling using DTPA	[61]
	Comparative study using various reducing agents for ^99m^Tc radiolabeling	Without drug	Direct radiolabeling using stannous chloride, sodium dithionite or sodium borohydride (reducing agent)	[62]
	Imaging of folate receptor overexpressed tumors	Folic acid	Direct radiolabeling using stannous chloride (reducing agent)	[63]
	Imaging of folate receptor or vascular endothelial growth factor receptor-2 (VEGFR-2) overexpressed tumors	Folic acid (FA) to target folate receptor and K237 (HTMYYHHYQHHL) peptide	Direct radiolabeling using stannous chloride (reducing agent)	[64]
	General biodistribution study	Gemcitabine	Direct radiolabeling using tricarbonyl kit	[65]
Lipid nanoparticles	Imaging of folate receptor overexpressed tumors	Cisplatin and folic acid	Direct radiolabeling using stannous chloride (reducing agent)	[68]
	Bone imaging	Methylene diphosphonate (MDP)	Direct radiolabeling using stannous chloride (reducing agent)	[69]
Liposomes nanoparticles	General biodistribution study	Without drug	Direct radiolabeling on the surface of nanoparticles	[71]
	General biodistribution study	Without drug	Chelator-based radiolabeling using 2-Iminothiolane and tricrbonyl kit	[72]
Oligomers	Human HepG2 cells targeting	Folic acid	Direct radiolabeling using stannous chloride (reducing agent)	[74]
Protein nanoparticles (Human Serum Albumin)	General biodistribution study	Bevacizumab	Direct radiolabeling using tricarbonyl kit	[77]
	General biodistribution study	HPMC, GMN2, GPM2 and GTM2 polymer coating	Direct radiolabeling using stannous chloride (reducing agent)	[78]

## 6. Conclusions and Future Perspectives

In preclinical and clinical research, the applications of nanoparticles are widely studied for both therapeutic applications and molecular imaging. The radiolabeling of nanoparticles is an accurate and sensitive method to understand their whole-body distribution and pharmacokinetics in both animal models and cancer patients. This review discussed various inorganic and organic nanoparticles, their surface modifications, radiolabeling strategies using ^99m^Tc, pharmacokinetics, and biodistribution results. Multiple strategies are available that can be used to obtain highly stable and efficiently radiolabeled nanoparticles with ^99m^Tc. The selection of radiolabeling methodology to obtain highly stable and pure ^99m^Tc radiolabeled nanoparticles depends entirely on the surface composition and structure of the nanoparticles. In this context, this mini-review will provide an excellent guideline to scientists and researchers working in nanomedicine research to choose the best strategy for the ^99m^Tc radiolabeling of their product.

## Figures and Tables

**Table 1:** Utilization, advantages, and limitations of imaging modalities.

Modality	Contrast Agent (Examples)	Spatial Resolution (mm)	Advantages	Limitations	Clinical Application
CT	Iodine; nanoparticles; barium; krypton	0.5–0.625 mm	Whole-body imaging available; high spatial resolution; short imaging time; unlimited depth penetration, inexpensive	Use of ionizing radiation; limited soft tissue contrast; molecular imaging not available; no real-time imaging	Yes
US	Microbubbles	0.04–0.1 (micro), 0.1–2 (clinical)	High sensitivity; non-ionizing radiation; real-time imaging; inexpensive, short acquisition time	Whole-body imaging not possible; limited depth penetration; limited contrast agents	Yes
Optical	Fluorescent dye; Nanoparticles	1–5 mm	High sensitivity; non-ionizing radiation; real time imaging; inexpensive, short acquisition time	Whole body imaging not possible; limited depth penetration; limited contrast agents	Yes
MRI	Gadolinium; iron oxide nanoparticles; manganese nanoparticles	0.01–0.1 (micro); 0.5–1.5 (clinical)	Non ionizing radiation; high spatial resolution; high soft tissue contrast; whole-body imaging possible; unlimited depth penetration	Expensive imaging; long acquisition time; low sensitivity	Yes
PET	Radioisotopes	1–2 (micro); 5–10 (clinical)	High sensitivity; whole-body imaging possible; unlimited depth penetration; quantitative imaging; can combine with other imaging technologies and therapy	Expensive imaging; low spatial resolution; long acquisition time; ionizing radiation exposure; need cyclotron or nuclear reactor	Yes
SPECT	Radioisotopes	0.5–2 (micro); 6–15 (clinical)	High sensitivity; whole-body imaging possible; unlimited depth penetration; quantitative imaging; can combine with other imaging technologies and therapy	Expensive imaging; low spatial resolution; long acquisition time; ionizing radiation exposure; need cyclotron or nuclear reactor	Yes

## References

[1] Sung H., Ferlay J., Siegel R.L., Laversanne M., Soerjomataram I., Jemal A., Bray F. (2021). Global cancer statistics 2020: GLOBOCAN estimates of incidence and mortality worldwide for 36 cancers in 185 countries. CA Cancer J. Clin..

[2] Tummers W.S., Miller S.E., Teraphongphom N.T., Gomez A., Steinberg I., Huland D.M., Hong S., Kothapalli S.R., Hasan A., Ertsey R. (2018). Intraoperative pancreatic cancer detection using tumor-specific multimodality molecular imaging. Ann. Surg. Oncol..

[3] Wu M., Shu J. (2018). Multimodal molecular imaging: Current status and future directions. Contrast Media Mol. Imaging..

[4] Hernandez Vargas S.H., Ghosh S.C., Azhdarinia A. (2019). New developments in dual-labeled molecular imaging agents. J. Nucl. Med..

[5] De La Pena H., Sharma A., Glicksman C., Joseph J., Subesinghe M., Traill Z., Verrill C., Sullivan M., Redgwell J., Bataillard E. (2017). No longer any role for routine follow-up chest x-rays in men with stage I germ cell cancer. Eur. J. Cancer.

[6] Al-Dhabyani W., Gomaa M., Khaled H., Fahmy A. (2020). Dataset of breast ultrasound images. Data Brief..

[7] Duan F., Hao D., Xu W., Zhong X., Luo T. (2019). Correlations of twist expression with pathological and computed tomography (CT) characteristics and prognosis of Non-Small Cell Lung Cancer (NSCLC). Med. Sci. Monit..

[8] Qi J., Chen C., Zhang X., Hu X., Ji S., Kwok R.T.K., Lam J.W.Y., Ding D., Tang B.Z. (2018). Light-driven transformable optical agent with adaptive functions for boosting cancer surgery outcomes. Nat. Commun..

[9] Stavrinides V., Giganti F., Trock B., Punwani S., Allen C., Kirkham A., Freeman A., Haider A., Ball R., McCartan N. (2020). Five-year outcomes of magnetic resonance imaging–based active surveillance for prostate cancer: A large cohort study. Eur. Urol..

[10] Lee S.J., Park H.J. (2020). Single photon emission computed tomography (SPECT) or positron emission tomography (PET) imaging for radiotherapy planning in patients with lung cancer: A meta-analysis. Sci. Rep..

[11] Corfield J., Perera M., Bolton D., Lawrentschuk N. (2018). ^68^Ga-prostate specific membrane antigen (PSMA) positron emission tomography (PET) for primary staging of high-risk prostate cancer: A systematic review. World J. Urol..

[12] Liu T., Wang S., Liu H., Meng B., Zhou F., He F., Shi X., Yang H. (2017). Detection of vertebral metastases: A meta-analysis comparing MRI, CT, PET, BS and BS with SPECT. J. Cancer Res. Clin. Oncol..

[13] Martí-Bonmatí L., Sopena R., Bartumeus P., Sopena P. (2010). Multimodality imaging techniques. Contrast Media Mol. Imaging.

[14] Lamb J., Holland J.P. (2018). Advanced methods for radiolabeling multimodality nanomedicines for SPECT/MRI and PET/MRI. J. Nucl. Med..

[15] Wang C., Fan W., Zhang Z., Wen Y., Xiong L., Chen X. (2019). Advanced nanotechnology leading the way to multimodal imaging-guided precision surgical therapy. Adv. Mater..

[16] Chen H., Gu Z., An H., Chen C., Chen J., Cui R., Chen S., Chen W., Chen X., Chen X. (2018). Precise nanomedicine for intelligent therapy of cancer. Sci. China Chem..

[17] Lombardo D., Kiselev M.A., Caccamo M.T. (2019). Smart nanoparticles for drug delivery application: Development of versatile nanocarrier platforms in biotechnology and nanomedicine. J. Nanomater..

[18] Janagam D.R., Wu L., Lowe T.L. (2017). Nanoparticles for drug delivery to the anterior segment of the eye. Adv. Drug Deliv. Rev..

[19] Salvanou E.A., Bouziotis P., Tsoukalas C. (2018). Radiolabeled nanoparticles in nuclear oncology. Adv. Drug Deliv. Rev..

[20] Chakravarty R., Bahadur J., Lohar S., Sarma H.D., Sen D., Mishra R., Chakraborty S., Dash A. (2019). Solid state synthesis of mesoporous alumina: A viable strategy for preparation of an advanced nanosorbent for ^99^Mo/^99^mTc generator technology. Microporous Mesoporous Mater..

[21] Duatti A. (2021). Review on ^99m^Tc radiopharmaceuticals with emphasis on new advancements. Nucl. Med. Biol..

[22] Reddy J.A., Xu L.C., Parker N., Vetzel M., Leamon C.P. (2004). Preclinical evaluation of ^99m^Tc-EC_20_ for imaging folate receptor–positive tumors. J. Nucl. Med..

[23] Carpenet H., Cuvillier A., Monteil J., Quelven I. (2015). Anti-CD_20_ immunoglobulin G radiolabeling with a ^99m^Tc-tricarbonyl core: *In vitro* and *in vivo* evaluations. PLoS ONE.

[24] Felber M., Alberto R. (2015). ^99m^Tc radiolabelling of Fe_3_O_4_-Au core-shell and Au–Fe_3_O_4_ dumbbell-like nanoparticles. Nanoscale.

[25] Putra A.R., Lestary E., Maskur M., Tahyan Y. (2020). Validation of [^99m^Tc] Tc-DTPA radiochemical testing method using one-system paper chromatography. J. Phys. Conf. Ser..

[26] Ling Y., Wei K., Luo Y., Gao X., Zhong S. (2011). Dual docetaxel/superparamagnetic iron oxide loaded nanoparticles for both targeting magnetic resonance imaging and cancer therapy. Biomaterials.

[27] Park S.H., Gwon H.J., Choi S.M. (2007). Preparation of ^99m^Tc-Labeled iron oxide nanoparticles for *in vivo* imaging in hyperthermia. Chem. Lett..

[28] Lee C.M., Jeong H.J., Kim E.M., Kim D.W., Lim S.T., Kim H.T., Park I.K., Jeong Y.Y., Kim J.W., Sohn M.H. (2009). Superparamagnetic iron oxide nanoparticles as a dual imaging probe for targeting hepatocytes *in vivo*. Magn. Reson. Med..

[29] Torres Martin de Rosales R., Tavaré R., Glaria A., Varma G., Protti A., Blower P.J. (2011). ^99m^Tc-bisphosphonate-iron oxide nanoparticle conjugates for dual-modality biomedical imaging. Bioconjug. Chem..

[30] Fatahian S., Shahbazi-Gahrouei D., Pouladian M., Yousefi M.H., Amiri G.R., Noori A. (2012). Biodistribution and toxicity assessment of radiolabeled and DMSA coated ferrite nanoparticles in mice. J. Radioanal. Nucl. Chem..

[31] Xue S., Zhang C., Yang Y., Zhang L., Cheng D., Zhang J., Shi H., Zhang Y. (2015). ^99m^Tc-labeled iron oxide nanoparticles for dual-contrast (T_1_/T_2_) magnetic resonance and dual-modality imaging of tumor angiogenesis. J. Biomed. Nanotechnol..

[32] Gao Z., Hou Y., Zeng J., Chen L., Liu C., Yang W., Gao M. (2017). Tumor microenvironment-triggered aggregation of antiphagocytosis ^99m^Tc-Labeled Fe_3_O_4_ nanoprobes for enhanced tumor imaging *in vivo*. Adv Mater..

[33] Psimadas D., Baldi G., Ravagli C., Franchini M.C., Locatelli E., Innocenti C., San Gregorio C., Loudos G. (2013). Comparison of the magnetic, radiolabeling, hyperthermic and biodistribution properties of hybrid nanoparticles bearing CoFe_2_O_4_ and Fe_3_O_4_ metal cores. Nanotechnology.

[34] Hainfeld J.F., Slatkin D.N., Smilowitz H.M. (2004). The use of gold nanoparticles to enhance radiotherapy in mice. Phys. Med. Biol..

[35] Mendoza-Sánchez A.N., Ferro-Flores G., Ocampo-García B.E., Morales-Avila E., de M. Ramírez F.D., De León-Rodríguez L.M., Santos-Cuevas C.L., Medina L.A., Rojas-Calderón E.L., Camacho-López M.A. (2010). Lys^3^-bombesin conjugated to ^99m^Tc-labelled gold nanoparticles for *in vivo* gastrin releasing peptide-receptor imaging. J. Biomed. Nanotechnol..

[36] Ocampo-García B.E., Ramírez F.D., Ferro-Flores G., De León-Rodríguez L.M., Santos-Cuevas C.L., Morales-Avila E., de Murphy C.A., Pedraza-López M., Medina L.A., Camacho-López M.A. (2011). ^99m^Tc-labelled gold nanoparticles capped with HYNIC-peptide/mannose for sentinel lymph node detection. Nucl. Med. Biol..

[37] Morales-Avila E., Ferro-Flores G., Ocampo-García B.E., De León-Rodríguez L.M., Santos-Cuevas C.L., García-Becerra R., Medina L.A., Gómez-Oliván L. (2011). Multimeric system of ^99m^Tc-labeled gold nanoparticles conjugated to c [RGDfK(C)] for molecular imaging of tumor α(v)β(3) expression. Bioconjug. Chem..

[38] Ocampo-García B., Ferro-Flores G., Morales-Avila E., de MaríaRamírez F. (2011). Kit for preparation of multimeric receptor-specific ^99m^Tc-radiopharmaceuticals based on gold nanoparticles. Nucl. Med. Commun..

[39] Jiménez-Mancilla N., Ferro-Flores G., Santos-Cuevas C., Ocampo-García B., Luna-Gutiérrez M., Azorín-Vega E., Isaac-Olivé K., Camacho-López M., Torres-García E. (2013). Multifunctional targeted therapy system based on ^99m^Tc/^177^Lu-labeled gold nanoparticles-Tat (49-57)-Lys^3^-bombesin internalized in nuclei of prostate cancer cells. J. Label. Comp. Radiopharm..

[40] Li X., Xiong Z., Xu X., Luo Y., Peng C., Shen M., Shi X. (2016). ^99m^Tc-labeled multifunctional low-generation dendrimer-entrapped gold nanoparticles for targeted SPECT/CT dual-mode imaging of tumors. ACS Appl. Mater. Interfaces.

[41] Kamal R., Chadha V.D., Dhawan D.K. (2018). Physiological uptake and retention of radiolabeled resveratrol loaded gold nanoparticles (^99m^Tc-Res-AuNP) in colon cancer tissue. Nanomedicine.

[42] Forner A., Llovet J.M., Bruix J. (2012). Hepatocellular carcinoma. Lancet.

[43] Zhou B., Wang R., Chen F., Zhao L., Wang P., Li X., Bányai I., Ouyang Q., Shi X., Shen M. (2018). ^99m^Tc-labeled RGD–polyethylenimine conjugates with entrapped gold nanoparticles in the cavities for dual-mode SPECT/CT imaging of hepatic carcinoma. ACS Appl. Mater. Interfaces.

[44] Xing Y., Zhu J., Zhao L., Xiong Z., Li Y., Wu S., Chand G., Shi X., Zhao J. (2018). SPECT/CT imaging of chemotherapy-induced tumor apoptosis using ^99m^Tc-labeled dendrimer-entrapped gold nanoparticles. Drug Deliv..

[45] Zhu J., Zhao L., Yang J., Chen L., Shi J., Zhao J., Shi X. (2019). ^99m^Tc-Labeled polyethylenimine-entrapped gold nanoparticles with pH-responsive charge conversion property for enhanced dual mode SPECT/CT imaging of cancer cells. Langmuir.

[46] Wen S., Zhao L., Zhao Q., Li D., Liu C., Yu Z., Shen M., Majoral J.P., Mignani S., Zhao J. (2017). A promising dual mode SPECT/CT imaging platform based on ^99m^Tc-labeled multifunctional dendrimer-entrapped gold nanoparticles. J. Mater. Chem. B.

[47] Tarn D., Ashley C.E., Xue M.I., Carnes E.C., Zink J.I., Brinker C.J. (2013). Mesoporous silica nanoparticle nanocarriers: Biofunctionality and biocompatibility. Acc. Chem. Res..

[48] Sá L.T., Pessoa C., Meira A.S., Da Silva M.I., Missailidis S., Santos-Oliveira R. (2012). Development of nanoaptamers using a mesoporous silica model labeled with ^99m^Tc for cancer targeting. Oncology.

[49] Tamba B.I., Dondas A., Leon M., Neagu A.N., Dodi G., Stefanescu C., Tijani A. (2015). Silica nanoparticles: Preparation, characterization and *in vitro*/*in vivo* biodistribution studies. Eur. J. Pharm. Sci..

[50] De Barros A.L.B., de Oliveira Ferraz K.S., Dantas T.C.S., Andrade G.F., Cardoso V.N., De Sousa E.M. (2015). Synthesis, characterization, and biodistribution studies of ^99m^Tc-labeled SBA-16 mesoporous silica nanoparticles. Mater. Sci. Eng. C Mater. Biol. Appl..

[51] Yamaguchi H., Tsuchimochi M., Hayama K., Kawase T., Tsubokawa N. (2016). Dual-labeled near-infrared/^99m^Tc imaging probes using PAMAM-coated silica nanoparticles for the imaging of HER2-expressing cancer cells. Int. J. Mol. Sci..

[52] Rainone P., Riva B., Belloli S., Sudati F., Ripamonti M., Verderio P., Colombo M., Colzani B., Gilardi M.C., Moresco R.M. (2017). Development of ^99m^Tc-radiolabeled nanosilica for targeted detection of HER2-positive breast cancer. Int. J. Nanomed..

[53] Rainone P., De Palma A., Sudati F., Roffia V., Rigamonti V., Salvioni L., Colombo M., Ripamonti M., Spinelli A.E., Mazza D. (2021). ^99m^Tc-radiolabeled silica nanocarriers for targeted detection and treatment of HER2-positive breast cancer. Int. J. Nanomed..

[54] Portilho F.L., Helal-Neto E., Cabezas S.S., Pinto S.R., Dos Santos S.N., Pozzo L., Sancenón F., Martínez-Máñez R., Santos-Oliveira R. (2018). Magnetic core mesoporous silica nanoparticles doped with dacarbazine and labelled with ^99m^Tc for early and differential detection of metastatic melanoma by single photon emission computed tomography. Artif. Cells Nanomed. Biotechnol..

[55] Wuillemin M.A., Reber M.J., Fox T., Spingler B., Brühwiler D., Alberto R., Braband H. (2019). Towards ^99m^Tc- and Re-based multifunctional silica platforms for theranostic applications. Inorganics.

[56] Gao H., Liu X., Tang W., Niu D., Zhou B., Zhang H., Liu W., Gu B., Zhou X., Zheng Y. (2016). ^99m^Tc-conjugated manganese-based mesoporous silica nanoparticles for SPECT, pH-responsive MRI and anti-cancer drug delivery. Nanoscale.

[57] Suchánková P., Kukleva E., Nykl E., Nykl P., Sakmár M., Vlk M., Kozempel J. (2020). Hydroxyapatite and titanium dioxide nanoparticles: Radiolabelling and *in vitro* stability of prospective theranostic nanocarriers for ^223^Ra and ^99m^Tc. Nanomaterials.

[58] Narmani A., Yavari K., Mohammadnejad J. (2017). Imaging, biodistribution and *in vitro* study of smart ^99m^Tc-PAMAM G4 dendrimer as novel nano-complex. Colloids Surf. B Biointerfaces.

[59] Ghoreishi S.M., Khalaj A., Sabzevari O., Badrzadeh L., Mohammadzadeh P., Mousavi Motlagh S.S., Bitarafan-Rajabi A., Shafiee Ardestani M. (2018). Technetium-99m chelator-free radiolabeling of specific glutamine tumor imaging nanoprobe: *In vitro* and *in vivo* evaluations. Int. J. Nanomed..

[60] Snehalatha M., Venugopal K., Saha R.N., Babbar A.K., Sharma R.K. (2008). Etoposide loaded PLGA and PCL nanoparticles II: Biodistribution and pharmacokinetics after radiolabeling with Tc-99m. Drug Deliv..

[61] Subramanian S., Dandekar P., Jain R., Pandey U., Samuel G., Hassan P.A., Patravale V., Venkatesh M. (2010). Technetium-99m–labeled poly (dl-Lactide-co-Glycolide) nanoparticles as an alternative for sentinel lymph node imaging. Cancer Biother. Radiopharm..

[62] Geskovski N., Kuzmanovska S., Simonoska Crcarevska M., Calis S., Dimchevska S., Petrusevska M., Zdravkovski P., Goracinova K. (2013). Comparative biodistribution studies of technetium-99m radiolabeled amphiphilic nanoparticles using three different reducing agents during the labeling procedure. J. Label. Comp. Radiopharm..

[63] Polyák A., Hajdu I., Bodnár M., Trencsényi G., Pöstényi Z., Haász V., Jánoki G., Jánoki G.A., Balogh L., Borbély J. (2013). ^99m^Tc-labelled nanosystem as tumour imaging agent for SPECT and SPECT/CT modalities. Int. J. Pharm..

[64] He Z., Zhang X., Huang J., Wu Y., Huang X., Chen J., Xia J., Jiang H., Ma J., Wu J. (2016). Immune activity and biodistribution of polypeptide K237 and folic acid conjugated amphiphilic PEG-PLGA copolymer nanoparticles radiolabeled with ^99m^Tc. Oncotarget.

[65] İçhedef Ç., Teksöz S., Çetin O., Aydın B., Sarıkavak İ., Parlak Y., Bilgin B.E. (2021). Design of ^99m^Tc radiolabeled gemcitabine polymeric nanoparticles as drug delivery system and *in vivo* evaluation. Mater. Chem. Phys..

[66] Fahy E., Cotter D., Sud M., Subramaniam S. (2011). Lipid classification, structures and tools. Biochim. Biophys. Acta.

[67] Puri A., Loomis K., Smith B., Lee J.H., Yavlovich A., Heldman E., Blumenthal R. (2009). Lipid-based nanoparticles as pharmaceutical drug carriers: From concepts to clinic. Crit. Rev. Ther. Drug Carr. Syst..

[68] Oumzil K., Khiati S., Camplo M., Koquely M., Chuttani K., Chaturvedi S., Mishra A.K., Barthélémy P. (2014). Nucleolipids as building blocks for the synthesis of ^99m^Tc-labeled nanoparticles functionalized with folic acid. New J. Chem..

[69] Mandiwana V., Kalombo L., Grobler A., Zeevaart J.R. (2018). ^99m^Tc-MDP as an imaging tool to evaluate the *in vivo* biodistribution of solid lipid nanoparticles. Appl. Radiat. Isot..

[70] Akbarzadeh A., Rezaei-Sadabady R., Davaran S., Joo S.W., Zarghami N., Hanifehpour Y., Samiei M., Kouhi M., Nejati-Koshki K. (2013). Liposome: Classification, preparation, and applications. Nanoscale Res. Lett..

[71] Lee C.M., Choi Y., Huh E.J., Lee K.Y., Song H.C., Sun M.J., Jeong H.J., Cho C.S., Bom H.S. (2005). Polyethylene glycol (PEG) modified ^99m^Tc-HMPAOliposome for improving blood circulation and biodistribution: The effect of the extent of pegylation. Cancer Biother. Radiopharm..

[72] Varga Z., Szigyártó I.C., Gyurkó I., Dóczi R., Horváth I., Máthé D., Szigeti K. (2017). Radiolabeling and quantitative *in vivo* SPECT/CT imaging study of liposomes using the novel iminothiolane-^99m^Tc-tricarbonyl complex. Contrast Media Mol. Imaging.

[73] Galzitskaya O.V. (2019). Oligomers are promising targets for drug development in the treatment of proteinopathies. Front. Mol. Neurosci..

[74] Liang L., Zhang X., Su X., Li J., Tian Y., Xue H., Xu H. (2018). ^99m^Tc-labeled oligomeric nanoparticles as potential agents for folate receptor-positive tumor targeting. J. Label. Comp. Radiopharm..

[75] Lohcharoenkal W., Wang L., Chen Y.C., Rojanasakul Y. (2014). Protein nanoparticles as drug delivery carriers for cancer therapy. Biomed. Res. Int..

[76] Jacob J., Haponiuk J.T., Thomas S., Gopi S. (2018). Biopolymer based nanomaterials in drug delivery systems: A review. Mater. Today Chem..

[77] Ramos-Membrive R., Erhard Á., de Redín I.L., Quincoces G., Collantes M., Ecay M., Irache J.M., Peñuelas I. (2020). *In vivo* SPECT-CT imaging and characterization of technetium-99m-labeled bevacizumab-loaded human serum albumin pegylated nanoparticles. J. Drug Deliv. Sci. Technol..

[78] de Arcocha-Torres M., Quincoces G., Martínez-López A.L., Erhard A., Collantes M., Martínez-Rodríguez I., Ecay M., Banzo I., Irache J.M., Peñuelas I. (2020). Preparation, radiolabeling with ^99m^Tc and ^67^Ga and biodistribution studies of albumin nanoparticles coated with polymers. Rev. Esp. Med. Nucl. Imagen Mol..

